# Supplementary material for: SHAPER-PND trial: clinical effectiveness protocol of a community singing intervention for postnatal depression
Source: BMJ Open. 2021 Nov 16;11(11):e052133. doi: 10.1136/bmjopen-2021-052133 (PMC8601068; doi:10.1136/bmjopen-2021-052133)
Supplement: Author's manuscript [file bmjopen-2021-052133.draft_revisions.pdf]

BMJ Open

BMJ Open is committed to open peer review. As part of this commitment we make the peer review history of every article we publish publicly available.

When an article is published we post the peer reviewers' comments and the authors' responses online. We also post the versions of the paper that were used during peer review. These are the versions that the peer review comments apply to.

The versions of the paper that follow are the versions that were submitted during the peer review process. They are not the versions of record or the final published versions. They should not be cited or distributed as the published version of this manuscript.

BMJ Open is an open access journal and the full, final, typeset and author-corrected version of record of the manuscript is available on our site with no access controls, subscription charges or pay-per-view fees (http://bmjopen.bmj.com).

If you have any questions on BMJ Open's open peer review process please email info.bmjopen@bmj.com

BMJ Open

The SHAPER-PND trial: clinical effectiveness protocol of a community singing intervention for postnatal depression

Journal:	*BMJ Open*
Manuscript ID	bmjopen-2021-052133
Article Type:	Protocol
Date Submitted by the Author:	08-Apr-2021
Complete List of Authors:	Estevao, Carolina; King's College London Institute of Psychiatry Psychology and Neuroscience, Department of Psychological Medicine<br>Bind, Rebecca; King’s College London, Department of Psychological Medicine<br>Fancourt, Daisy; University College London, Department of Behavioural Science and Health<br>Sawyer, Kristi; King’s College London, Department of Psychological Medicine<br>Dazzan, Paola; King's College London<br>Sevdalis, Nick; King's College London, Centre Implementation Science<br>Woods, Anthony; King’s College London, Department of Psychological Medicine<br>Crane, Nikki; King's College London<br>Rebecchini, Lavinia; King’s College London, Department of Psychological Medicine<br>Hazelgrove, Katie; King’s College London, Department of Psychological Medicine<br>Manoharan, Manonmani; South London and Maudsley NHS Foundation Trust<br>Burton, Alexandra; University College London, Department of Behavioural Science and Health<br>Dye, Hannah; Breathe Arts Health Research<br>Osborn, Tim; Breathe Arts Health Research<br>Davis, Rachel; King's College London, Centre for Implementation Science<br>Soukup, Tayana ; King's College London, Centre for Implementation Science<br>Bakolis, Ioannis; King's College London, Centre for Implementation Science; King's College London, Department of Biostatistics and Health Informatics<br>Healey, Andy; King's College London, Health Service and Population Research Department<br>Perkins, Rosie; Royal College of Music, Centre for Performance Science; Imperial College London, Faculty of Medicine<br>Pariante, Carmine; King's College London, Psychological Medicine
Keywords:	MENTAL HEALTH, PSYCHIATRY, Depression & mood disorders < PSYCHIATRY, IMMUNOLOGY

I, the Submitting Author has the right to grant and does grant on behalf of all authors of the Work (as defined in the below author licence), an exclusive licence and/or a non-exclusive licence for contributions from authors who are: i) UK Crown employees; ii) where BMJ has agreed a CC-BY licence shall apply, and/or iii) in accordance with the terms applicable for US Federal Government officers or employees acting as part of their official duties; on a worldwide, perpetual, irrevocable, royalty-free basis to BMJ Publishing Group Ltd ("BMJ") its licensees and where the relevant Journal is co-owned by BMJ to the co-owners of the Journal, to publish the Work in this journal and any other BMJ products and to exploit all rights, as set out in our licence.

The Submitting Author accepts and understands that any supply made under these terms is made by BMJ to the Submitting Author unless you are acting as an employee on behalf of your employer or a postgraduate student of an affiliated institution which is paying any applicable article publishing charge ("APC") for Open Access articles. Where the Submitting Author wishes to make the Work available on an Open Access basis (and intends to pay the relevant APC), the terms of reuse of such Open Access shall be governed by a Creative Commons licence – details of these licences and which Creative Commons licence will apply to this Work are set out in our licence referred to above.

Other than as permitted in any relevant BMJ Author's Self Archiving Policies, I confirm this Work has not been accepted for publication elsewhere, is not being considered for publication elsewhere and does not duplicate material already published. I confirm all authors consent to publication of this Work and authorise the granting of this licence.

For peer review only

BMJ OPEN

Title: The SHAPER-PND trial: clinical effectiveness protocol of a community singing intervention for postnatal depression

Authors: Carolina Estevao[1], Rebecca Bind[1], Daisy Fancourt[2], Kristi Sawyer[1], Paola Dazzan[1], Nick Sevdalis[3], Anthony Woods[1], Nikki Crane[4], Lavinia Rebecchini[1], Katie Hazelgrove[1], Manonmani Manoharan[5], Alexandra Burton[2], Hannah Dye[6], Tim Osborn[6], Rachel Davis[3], Tayana Soukup[3], Ioannis Bakolis[3,7], Andy Healey[8], Rosie Perkins[9,10], Carmine M. Pariante[1]

[1]Department of Psychological Medicine, Institute of Psychiatry, Psychology and Neuroscience, King's College London, 5 Cutcombe Rd, Brixton, London SE5 9RT, United Kingdom

[2]Department of Behavioural Science and Health, University College London, Gower Street, London, WC1E 6BT, United Kingdom

[3]Centre for Implementation Science, Health Service and Population Research Department, Institute of Psychiatry, Psychology & Neuroscience, 16 De Crespigny Park, Camberwell, London SE5 8AB, United Kingdom

[4]Culture team, King's College London, Somerset House East Wing, Strand WC2R 2LS, United Kingdom

[5]South London and Maudsley NHS Foundation Trust, Denmark Hill, London SE5 8AZ, United Kingdom

[6]Breathe Arts Health Research, The Clarence Centre, 6 St George's Circus, London SE1 6FE, United Kingdom

[7]Department of Biostatistics and Health Informatics, Institute of Psychiatry, Psychology & Neuroscience, 16 De Crespigny Park, Camberwell, London SE5 8AB, United Kingdom

[8]King's Health Economics, Health Service and Population Research Department, Institute of Psychiatry, Psychology & Neuroscience, 16 De Crespigny Park, Camberwell, London SE5 8AB, United Kingdom

[9]Centre for Performance Science, Royal College of Music, London, United Kingdom

[10]Faculty of Medicine, Imperial College London, London, United Kingdom

Corresponding author: Carolina Estevao, carolina.estevao@kcl.ac.uk

Keywords: arts intervention, randomised controlled trial, postnatal depression, mental health, singing

For peer review only

Word count: 3751 words

Abstract

Introduction: Postnatal depression (PND) affects approximately 13% of new mothers. Community-based activities are sought after by many mothers, especially mothers that prefer not to access pharmacological or psychological interventions. Singing has shown positive effects in maternal mood and mother-child bonding. The Scaling-Up Health-Arts Programmes: Implementation and Effectiveness Research – Postnatal Depression (SHAPER-PND) study will analyse the clinical and implementation effectiveness of 10-week singing sessions for PND in new mothers. This protocol paper will focus on the clinical effectiveness of this trial.

Methods and analysis: A total of 400 mothers and their babies will be recruited for this study. The intervention group will attend 10 weekly singing sessions held at community venues or online, facilitated by the arts organisation, Breathe Arts Health Research. A control group will be encouraged to attend non-singing sessions in the community or online for 10 weeks. A package of assessments will be collected from participants for clinical, mechanistic and implementation outcomes, at different stages of the trial. Clinical assessments will include questionnaires and interviews for demographics, mental health, and social measures, together with biological samples for measurement of stress markers; the study visits are at baseline, week 6 (mid-trial) and week 10 (end of trial), with follow ups at weeks 20 and 36.

Ethics and dissemination: Ethical approval has been granted by the London - West London & GTAC Research Ethics Committee, REC reference: 20/PR/0813.

Samples will be handled according to HTA regulations. All data will be handled in line with GDPR and other relevant regulations.

Informed consent will be collected from all research participants and stakeholders involved in the study.

Findings will be published in peer-reviewed journals and disseminated at national and international meetings. Participants will be made aware of the results of the study through a newsletter.

Article Summary

Strengths and limitations of this study

- Largest cohort of participants in a study examining singing in postnatal depression;

For peer review only

- Largest cohort studying biological measures in relation to an arts intervention for postnatal depression;
- Largest study to analyse the effect of an arts intervention on the mother-infant interaction;
- The state-of-the-art design will result in a unique package of clinical and implementation effectiveness on this intervention;
- There may be inconsistency throughout an intervention period if in-person sessions are switched online due to COVID-19 social distancing restrictions.

Introduction

Postnatal depression

Postnatal depression (PND) affects approximately 13% of new mothers, with symptoms including low mood, fatigue, anhedonia, insomnia and irritability [1,2]. Although pharmacological treatment has yielded positive results, these are hampered by low uptake and adherence, and while mother-infant psychotherapy has produced mixed results, poses similar challenges, including short-lived improvements [2–5].

Prior studies have found that PND carries implications for not only maternal health and wellbeing, but also for the mother-infant relationship [6]. A common finding is that, while interacting with their babies, depressed mothers spend more time in negative, withdrawn states [7,8] and exhibit reduced sensitivity [9,10], and in turn, their infants mirror this behaviour by disengaging and adopting a passive behavioural style [7]. Moreover, depressed mothers are more likely to report reduced feelings of attachment toward their infant [11] and are less likely to mentalize appropriately with their newborn [12]. Maternal sensitivity is highly predictive of infant secure attachment [13], which has long been linked to optimal developmental outcomes including mental health [14]; thus, intervention early-on to ameliorate the mother-infant interaction is warranted in order to protect offspring future development. However, while home-based interventions have been successful in improving PND, they show limited efficacy in helping the relationship itself [15]. Indeed, a recent combined psychoeducation and play intervention in a community setting was found to decrease dysfunctional parenting behaviours, but the intervention proved more effective in PND symptom reduction than in interaction improvement [17]; and a very small case study found that mother-infant painting sessions were successful in improving both the relationship and PND symptoms, though the sample size was only four dyads [18].

Despite these challenges in finding optimal treatment options, which can both alleviate maternal symptoms of PND and strengthen the mother-infant relationship, many mothers

For peer review only

continue to engage in community group activities, such as mother-infant play groups, as they are found to be effective at relaxing mothers, providing good sources of social interaction, decreasing the monotony of each day, and providing a sense of personal fulfilment for mothers [16]. Specifically, there is a growing body of evidence demonstrating the effects of community group singing on mental health [19,20] (see also the Melodies for Mums programme, described below). Singing to newborns is practised around the world, and research has demonstrated valuable benefits such as improving mother-infant interaction and reducing infant distress [21–23]. Listening to music during pregnancy is also associated with greater maternal wellbeing and reduced symptoms of PND in the first 3 months post-birth, and, additionally, daily singing to babies is associated with fewer symptoms of PND and increased maternal wellbeing, self-esteem and perceived mother-infant bond [24]. Consequently, there is a strong theoretical background for why singing could support mothers with PND. It is thought that postnatally depressed women may have difficulty with adherence to dyadic interventions given the nature and challenge of their symptoms [25]; however, no prior studies have assessed whether a community singing-based intervention may be able to both improve the quality of the developing relationship, as well as present as a more engaging intervention that mothers are likelier to complete.

Biological underpinning of singing sessions for postnatal depression

Three biological pathways are suggested to underlie the benefits of music engagement on mood: the glucocorticoid-modulated stress system, the inflammatory system, and the oxytocin system, which is also associated with maternal attachment and bonding. Of these, the hypothalamic-pituitary-adrenal (HPA) axis, of which the hormone cortisol is a main measure, is the most well-studied. Studies in various populations have given evidence for a reduction in salivary cortisol after a singing session [26–29], pointing to a consistent stress-reducing effect of singing. Interestingly, one of these studies also showed that singing sessions produced increases in the levels of cytokines such as IL-(interleukin-)17, IL-2, IL-4 and TNFα, which correlated with the observed reduction in cortisol [27]. The evidence surrounding the oxytocin system and music participation is more conflicted. Oxytocin is generally shown to increase as a result of singing [30,31], or even listening to music [32]. However, more recent studies have demonstrated a decrease in salivary oxytocin concentrations in certain circumstances, for example after choir singing, but not solo singing [28]. In addition, one study showed a decrease in oxytocin after singing sessions, which was associated with the increase in cytokines and reduction in cortisol, as described above [27]. This was proposed to be indicative of a general dampening of all stress-related signalling, in which oxytocin also plays a part, leading to an overall improved sense of wellbeing.

For peer review only

Studies investigating the interplay of these three biological systems in relation to musical engagement during the perinatal period are sparse. Indeed, only one study has investigated the biological effects of maternal singing groups [33], in which pregnant women were randomised to either music intervention, singing intervention, or control group. This study found a decrease in cortisol in both intervention groups, but that the effect was stronger in the singing group. Additionally, both musical intervention groups showed an increase in salivary oxytocin post-session. This is of particular interest given oxytocin's involvement in the mother-infant relationship [34], which may be strengthened in cases of mother-child biobehavioural synchrony, in which maternal and infant hormone levels covary [35].

The "Melodies for Mums" intervention

Melodies for Mums (M4M) is an intervention that was developed and tested as part of a collaboration between the Royal College of Music, Imperial College London and University College London (UK). M4M was tested in a three-arm randomised controlled trial (RCT) involving 134 mothers with PND (with an Edinburgh Postnatal Depression Scale (EPDS) score ≥ 10 at baseline, indicating depression), and compared the efficacy of community singing with a comparison group (10 weeks of creative play classes for dyads) and a wait-list control group (10 weeks of care-as-usual). This pilot study found that mothers with moderate-severe symptoms of PND, defined as an EPDS score of ≥13, who participated in the programme with their baby, had a significantly faster improvement in symptoms than mothers in usual care [36].

Following the pilot study, M4M was taken-on as a service by Breathe Arts Health Research (Breathe) across the boroughs of Lambeth and Southwark, in South London. However, there has since been no further research on the programme; thus, its effectiveness on a larger scale remains to be evaluated, as do the acceptability, appropriateness and feasibility of its delivery on a larger-scale, and its potential to be adopted and sustained as a cost-effective, beneficial service for mothers within national health services. In addition, there is no evidence on the effects of M4M on mother-infant interaction, and a detailed mechanistic investigation of the biological underpinning of any therapeutic effects is also missing. This paper will focus specifically on the protocol for a Wellcome-Trust-funded study aimed to deliver the clinical effectiveness and the biomarkers evaluation of M4M, the "Scaling-Up Health-Arts Programmes: Implementation and Effectiveness Research – Postnatal Depression" (SHAPER-PND) study. Information on the implementation effectiveness of M4M is discussed in a separate detailed protocol.

Methods and analysis

Aims and objectives

1) To determine the clinical effectiveness of the intervention in a larger sample size than previous studies (n= 400 women and their babies). This will allow us to ascertain whether the initial findings from the pilot study are replicable on a larger scale. To understand whether singing is an efficacious treatment for PND, including on mother-infant interaction, various psychological, social, and qualitative measures will be collected from women and their infants before, during, and after the intervention period.

2) To ascertain the biological mechanisms by which postnatal singing groups putatively improve maternal mood and mother-infant relationships. This will further the current understanding of the positive effects of the intervention and provide objectively measurable outcomes. Maternal and infant samples will be collected to measure cortisol and oxytocin, plus maternal inflammatory markers.

Primary objective

1. Does community singing improve symptoms of PND?

Participants will complete the EPDS prior to the intervention (baseline) to ascertain the severity of their PND. The EPDS will be administered again at various timepoints throughout and following the intervention period (weeks 6, 10, 20 and 36) to evaluate whether the intervention effectively reduces symptoms of PND. The primary outcome of this study is to understand whether severity of symptoms of PND on the EPDS is reduced between baseline and week 10 (end of intervention).

Secondary Objectives

2. Does community singing improve further aspects of maternal mental health?

Further measures of mental health will be administered at baseline and again at weeks 6, 10, 20 and 36 to assess whether singing ameliorates other aspects of mental health. As such, the Beck Depression Inventory (BDI) and the Hamilton Depression Rating Scale (HAM-D) will be administered to assess further traits of depression; the State-Trait Anxiety Inventory (STAI) and Perceived Stress Scale (PSS) will be administered to evaluate levels of anxiety and stress; and the Office for National Statistics Wellbeing Scale (ONS) will be used to gather information on general satisfaction and wellbeing.

3. Does community singing facilitate the mother-infant relationship?

The study will evaluate aspects of the dyad at baseline, and again throughout the intervention, to establish whether singing is an appropriate treatment for difficulties in the forming of relationships with infants among women with PND. Specifically, mothers will complete the

For peer review only - http://bmjopen.bmj.com/site/about/guidelines.xhtml

Maternal Postnatal Attachment Scale (MPAS) and the Parental Reflective Functioning Questionnaire (PRFQ) at baseline and weeks 6, 10, 20 and 36, to report on their subjective experience of the relationship; and, furthermore, dyads will be filmed at baseline and weeks 10 and 36 and rated for the quality of the interaction according to the Crittenden Child-Adult Relationship Experimental Index (CCI), also known as the CARE-Index.

4. Does community singing reduce loneliness and improve social support?

To evaluate whether community singing is an effective way to decrease feelings of loneliness and increase perceived social support in women with PND, participants will be asked to complete the UCLA Loneliness Scale, the Short General Self-Efficacy Scale (GSE-6), and the Multidimensional Scale of Perceived Social Support (MSPSS) at baseline and again throughout the intervention period at weeks 6, 10, 20 and 36.

5. Are there certain biological mechanisms which underpin psychological change?

Biological samples will be taken from mothers and their infants throughout the course of the intervention. The dyad will each provide diurnal saliva samples before and after the 10-week intervention in order to assess for changes in their diurnal cortisol output. Furthermore, we will collect a saliva sample from the dyad before and after their first, sixth and tenth sessions to evaluate changes in oxytocin, cortisol, and inflammatory markers from an individual music session. Finally, to assess overall cortisol output over the 10-week period, mothers will provide a hair sample at the end of the intervention.

6. Does community singing affect the lived experience of women with PND?

Focus groups will be conducted with women who have participated in the intervention to identify how the singing sessions affect the lived experience of mothers with PND, and their reported mechanisms of effect. More specifically, the focus groups will capture general feedback from sessions and experiences from mothers in the groups.

7. How does community singing interact with PND in women with particular risk factors for PND?

Individual or small-group semi-structured interviews with three sub-groups of women self-reporting particular risk factors for PND (traumatic birth, adverse childhood experiences, social isolation/loneliness) will be conducted. These interviews will focus in-depth on the phenomenology of PND and how singing intersects with the specific context of PND among these sub-groups.

Data collection plan

For peer review only

In order to address the objectives within the clinical effectiveness arm of the study, data will be collected according to the data collection timeline in Table 1.

Table 1 Clinical effectiveness data collection timeline.

**Study objective**	**Type of measure**	**Measures**	**Timepoint collected**	**Type of data**
1	Mental health	Edinburgh Postnatal Depression Scale (EPDS)	Baseline, weeks 1, 6, 10, 20 & 36	Quantitative
2	Mental health	Hamilton Depression Rating Scale (HAM-D)	Weeks 1 & 10	Quantitative
		Beck Depression Inventory (BDI), State Trait Anxiety Inventory (STAI), Perceived Stress Scale (PSS); Office for National Statistics Wellbeing Scale (ONS)	Weeks 1, 6, 10, 20 & 36	
3	Social	Maternal Postpartum Attachment Scale (MPAS), Parental Reflective Functioning Questionnaire (PRFQ)	Weeks 1, 6, 10, 20 & 36	Quantitative
		Crittenden Child-Adult Relationship (CCI CARE-Index)[1]	Baseline, weeks 10 & 36	
4	Social	UCLA Loneliness Scale, Short General Self-Efficacy Scale (GSE-6), Multidimensional Scale of Perceived Social Support (MSPSS)	Weeks 1, 6, 10, 20 & 36	Quantitative
		Diurnal cortisol saliva samples[1]	Baseline & week 10	

For peer review only

5	Biological	Pre- and post-session saliva samples[1]	Weeks 1, 6, 10 (at sessions)	Quantitative
		Hair samples	Week 10	
6	Qualitative	Focus groups with intervention groups to capture feedback from sessions and experiences from mothers in the groups, focusing on the lived experience of PND	Week 10	Qualitative
7	Qualitative	Semi-structured interviews with a sub sample of the intervention group of women self-reporting particular risk factors for PND (partially informed by demographic questionnaires)	Weeks 10-20, informed by information collected at baseline	Qualitative

[1] Collected from mothers and infants

Design

Scaling-Up Health-Arts Programmes: Implementation and Effectiveness Research (SHAPER) is a multi-disciplinary research programme funded by the Wellcome Trust that is hosted by King's College London (Sponsor) and University College London. The SHAPER-PND study described here is Hybrid Type II Effectiveness-Implementation randomised-controlled trial, where equal focus is placed on the effectiveness of the intervention and the effectiveness of its implementation [37].

Study Flowchart

An overview of the study timelines and procedures has been summarised in the flowchart below (

Figure 1):

For peer review only

Setting

This is a multi-centre trial that will be run in locations across London (primarily in the South London boroughs of Lewisham, Lambeth and Southwark), specifically in children's or community venues or online. These venues will be thoroughly safety- and risk-assessed in light of COVID-19 and adherence to any COVID-secure measures will be closely monitored by Breathe.

In order to enrol a sufficiently large sample of women, there will be 12 blocks of the 10-week singing programme in total: 10 intervention blocks will be for the experimental group, and 2 blocks will be for women in the wait-list control group that wish to take part in the singing sessions. The 2 wait-list blocks will be offered singing sessions after a woman has completed the first 10 weeks of the study and no data will be collected regarding these singing sessions, apart from the control follow-up data that this group is expected to provide.

In the event that one or several women need to self-isolate due to confirmed or suspected exposure to COVID-19, the sessions will be delivered to her/them online via Zoom. This will allow for the sessions to still be conducted in person with the remaining women.

In the event of the artist or the Breathe officer having to self-isolate, a replacement Breathe-trained artist will be ready to step-in and deliver the sessions. The same will apply to Breathe staff; there will be another staff member available to be present at the sessions.

Participants

Study participants will be new mothers exhibiting symptoms of PND, who meet criteria for a current major depressive episode, and have babies aged 0-9 months.

Inclusion Criteria: Women aged 18 or older; satisfactory understanding of English; women with infants aged 0-9 months; women with PND, who meet criteria for a current major depressive episode according to the Structured Clinical Interview for DSM-IV Disorders (SCID), in addition to a score of ≥10 on the EPDS. Women will require access to an internet-connected device (mobile phone, tablet, computer or laptop) to allow completion of assessments and participation in the singing sessions in the event they go online.

Exclusion Criteria: The participant may not enter the study if her baby is outside of the age-range specified; if she is unable to give informed consent.

Number of participants and power analysis

The pilot RCT that informed this study showed that depressive symptoms (EPDS scores) improved in both singing and control women by the end of treatment (week 10), but the singing

For peer review only - http://bmjopen.bmj.com/site/about/guidelines.xhtml

group had a numerically larger improvement (-5.2 (SD=2.8)) than the care-as-usual group (-4.25 (SD=3.2), effect size=0.32)) [36]. To have power of 80% (at p<0.05, two-tailed) to detect the same effect size difference, randomising subjects 2:1, 232 subjects will be required in the intervention and 116 in the control group (total=348). In total, 400 mothers and their babies will be recruited to compensate for a 12% drop-out rate.

Participants will be recruited in blocks of 40-50 women and their babies, screened and assessed, prior to a 2:1 allocation randomisation (dyads will be allocated the same group). This process will be repeated 12 times, aiming to reach 400 dyads.

Recruitment process

The recruitment period will last two years (2021-2022). All potential participants will initially be directed to a pre-screening online form that includes an EPDS. If the EPDS score is < 10, a participant will be notified that she is not currently eligible to participate and will be signposted to other support services within the community (e.g., talking therapies, mother-baby groups, baby activity groups, etc). If a woman's score is ≥ 10, she will be notified that she is potentially eligible and will proceed straight to the second phase of screening through a home (or Zoom) visit. The participant information sheet (PIS) for the trial will be sent once the baseline assessment is booked.

When a new round of 10 classes becomes available, researchers will arrange assessments with potential participants to undertake the full screening interview against the inclusion/exclusion criteria. If the participant is found non-eligible, she will be signposted to other support services within the community.

The singing group

M4M is a 10-week intervention for mothers with PND. The programme will be delivered to groups of 8–12 mothers in weekly sessions lasting one hour. Mothers will start a block of 10-week classes together and continue with the same group and leader for the duration of the course. Classes are free to attend and will take place in Children's Centres or other community venues (or online via Zoom). Mothers will attend with their babies and will be invited to sit in a socially distanced circle on the floor surrounded by soft play cushions and mats. Classes will start with welcome songs, introducing the babies and mothers to one another, and then involve a range of singing and music activities. Mothers will be required to respect social distancing guidelines and keep their babies within their reach to avoid physical contact with other mothers or their babies.

Some songs will be accompanied by maracas, drums, hand chimes and other simple instruments that the mothers and babies can play together. Instruments such as guitar and

For peer review only

ukulele will also be used for a small number of songs. Instruments will not be shared between the participants and will be disinfected before and after the singing sessions with a COVID-19 validated disinfectant.

Mothers will also work to write some of their own songs over the weeks, developing lyrics together about their babies or experiences of motherhood and creating simple melodies. Recordings of the group singing the songs together will be made and uploaded to private online platforms or onto CDs for the mothers to listen to at home. Classes will be led by professional workshop leaders trained by Breathe, with support of assistants.

The control group

The control group will be an 'active' control. During the first 10 weeks (during the study period), mothers in the control group will receive details of other non-music classes available to them in the community (either in-person or online, depending on the programs available at the time and government guidelines) and will receive the same schedule of texts and phone calls to encourage them to join these activities. They will still be seen by the researchers to collect clinical measures and biological samples (including the pre-post saliva samples) and to monitor engagement in other activities. Following the first 10 weeks, the mothers in the control group will be offered a place on the singing programme, but these data will not be part of the study, and they will not join groups with other women who are in the study.

Patient and public involvement

Participants from previous sessions delivered by Breathe in the community have provided valuable feedback and insights into the sessions' delivery, content and schedule. These were gathered through focus groups and workshops where mothers and others involved in the singing sessions were able to share their experience of the sessions. This body of work allowed the refinement of the sessions for the intervention to be more suitable for the population.

A previous RCT was designed and delivered by two of this study's co-authors (DF, RP), along with Breathe [36]. The RCT analysed the clinical efficacy of the intervention on a smaller scale and explored the experiences of participants receiving and delivering the intervention, which ultimately informed the design of this SHAPER-PND study.

Data Analyses

Quantitative analyses

For peer review only

The first stage of analyses will be a descriptive model of the data to assess completeness of data. Mother-level baseline variables will be described both overall and by randomised groups. Patterns of missing data will be described. The primary outcome will be analysed using linear mixed models to model the change in total score of EPDS at 6-, 10-, 20- and 36-weeks post randomisation. A three-level hierarchical model will be employed with all-time points included as repeated measures in the model (baseline and 6-, 10-, 20- and 36-weeks post randomisation) to improve power and take into account clustering of the observation at mother's level. These models use maximum likelihood estimation and thus allow for missing outcome data under the missing at random (MAR) assumption. Associations between post randomisation variables and missingness will be dealt with by multiple imputation, again under the MAR assumption. Departures from this assumption will be assessed with a sensitivity analysis. Secondary outcomes (e.g., PSS, HAM-D) will be assessed with a similar methodology for the primary outcomes, using generalised linear mixed models depending on the type of outcome (normal, binary and count). The questionnaires to be used have validated methods of scoring, and the scores will be analysed as described. All analyses will be conducted in STATA V.15.1.

Qualitative analyses

Data from the focus groups will be analysed using Thematic Analysis [38], to understand the lived experience of PND and how this relates to experiences in the singing group. Data from the individual/small-group interviews with sub-groups of women self-reporting particular risk factors for PND (traumatic birth, adverse childhood experiences, and social isolation/loneliness) will be analysed using Interpretative Phenomenological Analysis (IPA) [39] to focus in-depth on how singing intersects with the specific context of PND among women in these sub-groups.

Conclusion

To our knowledge, this study is the largest scale effectiveness and implementation combined trial on community singing groups and postnatal depression in new mothers to date. By combining measures of clinical and implementation effectiveness, the SHAPER-PND study offers a unique contribution to the evidence base and will result in robust and readily applicable insights to how best mothers with PNSD can be best supported and whether an arts-driven programme such as M4M is effective and scalable. Should the trial replicate the prior smaller scale efficacy study [36] and produce positive findings, the combined clinical and implementation evaluations will create a strong case for embedding the intervention into current clinical pathways.

For peer review only

Contributorship statement

All authors listed have contributed to the conception and design of the protocol and this manuscript. All authors have been involved in the drafting of the manuscript and have individually approved the version of the work published.

Competing interests

NS is the director of the London Safety and Training Solutions Ltd, which offers training in patient safety, implementation solutions and human factors to healthcare organisations.

DF is a non-executive board director for Breathe Arts Health Research but she receives no financial compensation for her involvement.

TS received funding for training cancer multidisciplinary teams in the assessment and quality improvement methods in the United Kingdom. TS serves as a consultant to F. Hoffmann-La Roche Diagnostics providing advisory research services in relation to innovations for cancer multidisciplinary teams and their care planning meetings in the United States of America.

CMP has received research and consultation funding from Boehringer Ingelheim and Johnson & Johnson for research on depression and inflammation, and by a Wellcome Trust strategy award to the Neuroimmunology of Mood Disorders and Alzheimer's Disease (NIMA) Consortium (104025), which is also funded by Janssen, GlaxoSmithKline, Lundbeck and Pfizer; the work presented in this paper is unrelated to this funding.

The other authors have no conflicts of interest to declare.

Funding Statement

This trial is part of the SHAPER programme, a Scaling-up Health-Arts Programme to scale up arts interventions. This programme is funded by the Wellcome Trust (award reference 219425/Z/19/Z). This work is additionally supported by the National Institute for Health Research (NIHR) Biomedical Research Centre at South London and Maudsley NHS Foundation Trust and King's College London and by a NIHR Senior Investigator to CMP.

NS, IB and AH' research is supported by the National Institute for Health Research (NIHR) Applied Research Collaboration (ARC) South London at King's College Hospital NHS Foundation Trust. NS and AH are members of King's Improvement Science, which offers co-funding to the NIHR ARC South London and comprises a specialist team of improvement scientists and senior researchers based at King's College London. Its work is funded by King's

For peer review only

Health Partners (Guy's and St Thomas' NHS Foundation Trust, King's College Hospital NHS Foundation Trust, King's College London and South London and Maudsley NHS Foundation Trust), Guy's and St Thomas' Charity and the Maudsley Charity. The views expressed in this publication are those of the authors and not necessarily those of the Wellcome Trust, the NIHR, or the Department of Health and Social Care.

RD is supported by the National Institute for Health Research (NIHR) Applied Research Collaboration: South London, at King's College Hospital NHS Foundation Trust. The views expressed in this publication are those of the author(s) and not necessarily those of the NHS, NIHR or the Department of Health and Social Care.

Data sharing statement: Not applicable, this manuscript does not contain any data.

References

1 Boath E, Henshaw C. The treatment of postnatal depression: A comprehensive literature review. *Journal of Reproductive and Infant Psychology* 2001;**19**:215–48. doi:10.1080/02646830120073224

2 Morrell C, Warner R, Slade P, *et al.* Psychological interventions for postnatal depression: cluster randomised trial and economic evaluation. The PoNDER trial. *Health Technol Assess* 2009;**13**. doi:10.3310/hta13300

3 Bledsoe SE, Grote NK. Treating Depression During Pregnancy and the Postpartum: A Preliminary Meta-Analysis. *Research on Social Work Practice* 2006;**16**:109–20. doi:10.1177/1049731505282202

4 Cooper PJ, Murray L, Wilson A, *et al.* Controlled trial of the short- and long-term effect of psychological treatment of post-partum depression 1. Impact on maternal mood. *BJP* 2003;**182**:412–9. doi:10.1192/bjp.182.5.412

5 Milgrom J, Negri LM, Gemmill AW, *et al.* A randomized controlled trial of psychological interventions for postnatal depression. *Br J Clin Psychol* 2005;**44**:529–42. doi:10.1348/014466505X34200

6 Murray L, Fearon P, Cooper P. Postnatal depression, mother-infant interactions, and child development: Prospects for screening and treatment. In: *Identifying perinatal depression and anxiety: Evidence-based practice in screening, psychosocial assessment, and management.* Wiley-Blackwell 2015. 139–64.

For peer review only

7 Field T, Healy BT, Goldstein S, *et al.* Behavior-state matching and synchrony in mother-infant interactions of nondepressed versus depressed dyads. *Developmental Psychology* 1990;**26**:7–14. doi:10.1037/0012-1649.26.1.7

8 Field T. Maternal Depression Effects on Infants and Early Interventions. *Preventive Medicine* 1998;**27**:200–3. doi:10.1006/pmed.1998.0293

9 Field T. Postpartum depression effects on early interactions, parenting, and safety practices: A review. *Infant Behavior and Development* 2010;**33**:1–6. doi:10.1016/j.infbeh.2009.10.005

10 Murray L, Fiori-Cowley A, Hooper R, *et al.* The impact of postnatal depression and associated adversity on early mother-infant interactions and later infant outcome. *Child Dev* 1996;**67**:2512–26.

11 Moehler E, Brunner R, Wiebel A, *et al.* Maternal depressive symptoms in the postnatal period are associated with long-term impairment of mother–child bonding. *Arch Womens Ment Health* 2006;**9**:273–8. doi:10.1007/s00737-006-0149-5

12 Pawlby S, Fernyhough C, Meins E, *et al.* Mind-mindedness and maternal responsiveness in infant–mother interactions in mothers with severe mental illness. *Psychol Med* 2010;**40**:1861–9. doi:10.1017/S0033291709992340

13 Campbell SB, Brownell CA, Hungerford A, *et al.* The course of maternal depressive symptoms and maternal sensitivity as predictors of attachment security at 36 months. *Develop Psychopathol* 2004;**16**. doi:10.1017/S0954579404044499

14 DeKlyen M, Greenberg M. Attachment and psychopathology in childhood. In: *Handbook of attachment: Theory, research, and clinical applications*. The Guilford Press 2008. 637–65.

15 Gelfand DM, Teti DM, Seiner SA, *et al.* Helping mothers fight depression: Evaluation of a home-based intervention program for depressed mothers and their infants. *Journal of Clinical Child Psychology* 1996;**25**:406–22. doi:10.1207/s15374424jccp2504_6

16 Feeley N, Bell L, Hayton B, *et al.* Care for Postpartum Depression: What Do Women and Their Partners Prefer? *Perspect Psychiatr Care* 2016;**52**:120–30. doi:10.1111/ppc.12107

For peer review only

17 Ericksen J, Loughlin E, Holt C, *et al.* A therapeutic playgroup for depressed mothers and their infants: feasibility study and pilot randomized trial of community hugs. *Infant Ment Health J* 2018;**39**:396–409. doi:10.1002/imhj.21723

18 Arroyo C, Fowler N. Before and after: A mother and infant painting group. *International Journal of Art Therapy* 2013;**18**:98–112. doi:10.1080/17454832.2013.844183

19 Clift S, Morrison I. Group singing fosters mental health and wellbeing: findings from the East Kent "singing for health" network project. *Mental Health Social Inclusion* 2011;**15**:88–97. doi:10.1108/20428301111140930

20 Coulton S, Clift S, Skingley A, *et al.* Effectiveness and cost-effectiveness of community singing on mental health-related quality of life of older people: randomised controlled trial. *Br J Psychiatry* 2015;**207**:250–5. doi:10.1192/bjp.bp.113.129908

21 Mualem O, Klein PS. The communicative characteristics of musical interactions compared with play interactions between mothers and their one-year-old infants. *Early Child Development and Care* 2013;**183**:899–915. doi:10.1080/03004430.2012.688824

22 Nakata T, Trehub SE. Infants' responsiveness to maternal speech and singing. *Infant Behavior and Development* 2004;**27**:455–64. doi:10.1016/j.infbeh.2004.03.002

23 Vlismas W, Malloch S, Burnham D. The effects of music and movement on mother–infant interactions. *Early Child Development and Care* 2013;**183**:1669–88. doi:10.1080/03004430.2012.746968

24 Fancourt D, Perkins R. Associations between singing to babies and symptoms of postnatal depression, wellbeing, self-esteem and mother-infant bond. *Public Health* 2017;**145**:149–52. doi:10.1016/j.puhe.2017.01.016

25 Nylen KJ, Moran TE, Franklin CL, *et al.* Maternal depression: A review of relevant treatment approaches for mothers and infants. *Infant Ment Health J* 2006;**27**:327–43. doi:10.1002/imhj.20095

26 Fancourt D, Aufegger L, Williamon A. Low-stress and high-stress singing have contrasting effects on glucocorticoid response. *Front Psychol* 2015;**6**. doi:10.3389/fpsyg.2015.01242

27 Fancourt D, Williamon A, Carvalho LA, *et al.* Singing modulates mood, stress, cortisol, cytokine and neuropeptide activity in cancer patients and carers. *ecancer* 2016;**10**. doi:10.3332/ecancer.2016.631

For peer review only

28 Schladt TM, Nordmann GC, Emilius R, *et al.* Choir versus Solo Singing: Effects on Mood, and Salivary Oxytocin and Cortisol Concentrations. *Front Hum Neurosci* 2017;**11**:430. doi:10.3389/fnhum.2017.00430

29 Grebosz-Haring K, Thun-Hohenstein L. Effects of group singing versus group music listening on hospitalized children and adolescents with mental disorders: A pilot study. *Heliyon* 2018;**4**:e01014. doi:10.1016/j.heliyon.2018.e01014

30 Grape C, Sandgren M, Hansson L-O, *et al.* Does singing promote well-being?: An empirical study of professional and amateur singers during a singing lesson. *Integrative Physiological & Behavioral Science* 2002;**38**:65–74. doi:10.1007/BF02734261

31 Kreutz G. Does singing promote social bonding? *Music and Medicine* 2014;**6**:51–60.

32 Nilsson U. Soothing music can increase oxytocin levels during bed rest after open-heart surgery: a randomised control trial. *Journal of Clinical Nursing* 2009;**18**:2153–61. doi:10.1111/j.1365-2702.2008.02718.x

33 Wulff V, Hepp P, Wolf OT, *et al.* The effects of a music and singing intervention during pregnancy on maternal well-being and mother–infant bonding: a randomised, controlled study. *Arch Gynecol Obstet* Published Online First: 10 August 2020. doi:10.1007/s00404-020-05727-8

34 Galbally M, Lewis AJ, van IJzendoorn M, *et al.* The Role of Oxytocin in Mother-Infant Relations: A Systematic Review of Human Studies: *Harvard Review of Psychiatry* 2011;**19**:1–14. doi:10.3109/10673229.2011.549771

35 Feldman R. Oxytocin and social affiliation in humans. *Hormones and Behavior* 2012;**61**:380–91. doi:10.1016/j.yhbeh.2012.01.008

36 Fancourt D, Perkins R. Effect of singing interventions on symptoms of postnatal depression: three-arm randomised controlled trial. *The British Journal of Psychiatry* 2018;**212**:119–21. doi:10.1192/bjp.2017.29

37 Curran GM, Bauer M, Mittman B, *et al.* Effectiveness-implementation hybrid designs: combining elements of clinical effectiveness and implementation research to enhance public health impact. *Med Care* 2012;**50**:217–26. doi:10.1097/MLR.0b013e3182408812

38 Smith JA. *Qualitative Psychology: A Practical Guide to Research Methods*. SAGE 2015.

For peer review only

39 Smith JA, Shinebourne P. Interpretative phenomenological analysis. In: *APA handbook of research methods in psychology, Vol 2: Research designs: Quantitative, qualitative, neuropsychological, and biological*. Washington, DC, US: : American Psychological Association 2012. 73–82. doi:10.1037/13620-005

Figure legend:

Figure 1 SHAPER-PND study flowchart.

For peer review only

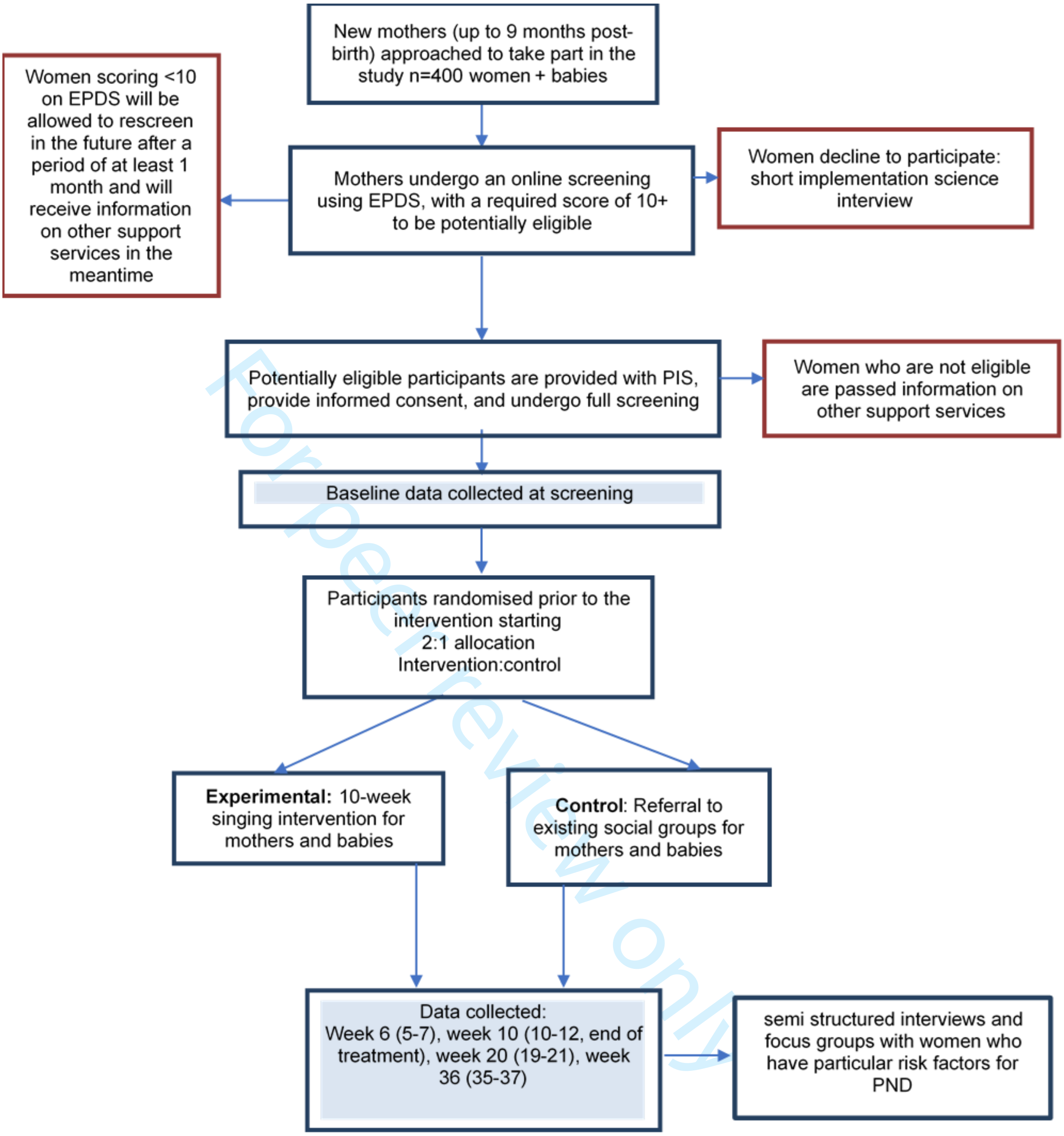

For peer review only

Reporting checklist for protocol of a clinical trial.

Based on the SPIRIT guidelines.

Instructions to authors

Complete this checklist by entering the page numbers from your manuscript where readers will find each of the items listed below.

Your article may not currently address all the items on the checklist. Please modify your text to include the missing information. If you are certain that an item does not apply, please write "n/a" and provide a short explanation.

Upload your completed checklist as an extra file when you submit to a journal.

In your methods section, say that you used the SPIRITreporting guidelines, and cite them as:

Chan A-W, Tetzlaff JM, Gøtzsche PC, Altman DG, Mann H, Berlin J, Dickersin K, Hróbjartsson A, Schulz KF, Parulekar WR, Krleža-Jerić K, Laupacis A, Moher D. SPIRIT 2013 Explanation and Elaboration: Guidance for protocols of clinical trials. BMJ. 2013;346:e7586

		Reporting Item	Page Number
**Administrative information**			
Title	#1	Descriptive title identifying the study design, population, interventions, and, if applicable, trial acronym	1
Trial registration	#2a	Trial identifier and registry name. If not yet registered, name of intended registry	1
Trial registration: data set	#2b	All items from the World Health Organization Trial Registration Data Set	1-14
Protocol version	#3	Date and version identifier	1
Funding	#4	Sources and types of financial, material, and other support	19-20
Roles and responsibilities: contributorship	#5a	Names, affiliations, and roles of protocol contributors	1

For peer review only

Roles and responsibilities: sponsor contact information	#5b	Name and contact information for the trial sponsor	9
Roles and responsibilities: sponsor and funder	#5c	Role of study sponsor and funders, if any, in study design; collection, management, analysis, and interpretation of data; writing of the report; and the decision to submit the report for publication, including whether they will have ultimate authority over any of these activities	9
Roles and responsibilities: committees	#5d	Composition, roles, and responsibilities of the coordinating centre, steering committee, endpoint adjudication committee, data management team, and other individuals or groups overseeing the trial, if applicable (see Item 21a for data monitoring committee)	N/A
**Introduction**			
Background and rationale	#6a	Description of research question and justification for undertaking the trial, including summary of relevant studies (published and unpublished) examining benefits and harms for each intervention	3-5
Background and rationale: choice of comparators	#6b	Explanation for choice of comparators	9
Objectives	#7	Specific objectives or hypotheses	6-7
Trial design	#8	Description of trial design including type of trial (eg, parallel group, crossover, factorial, single group), allocation ratio, and framework (eg, superiority, equivalence, non-inferiority, exploratory)	9
**Methods: Participants, interventions, and outcomes**			
Study setting	#9	Description of study settings (eg, community clinic, academic hospital) and list of countries where data will be collected. Reference to where list of study sites can be obtained	11
Eligibility criteria	#10	Inclusion and exclusion criteria for participants. If applicable, eligibility criteria for study centres and individuals who will	12

For peer review only

		perform the interventions (eg, surgeons, psychotherapists)	
Interventions: description	#11a	Interventions for each group with sufficient detail to allow replication, including how and when they will be administered	5
Interventions: modifications	#11b	Criteria for discontinuing or modifying allocated interventions for a given trial participant (eg, drug dose change in response to harms, participant request, or improving / worsening disease)	N/A
Interventions: adherance	#11c	Strategies to improve adherence to intervention protocols, and any procedures for monitoring adherence (eg, drug tablet return; laboratory tests)	N/A
Interventions: concomitant care	#11d	Relevant concomitant care and interventions that are permitted or prohibited during the trial	N/A
Outcomes	#12	Primary, secondary, and other outcomes, including the specific measurement variable (eg, systolic blood pressure), analysis metric (eg, change from baseline, final value, time to event), method of aggregation (eg, median, proportion), and time point for each outcome. Explanation of the clinical relevance of chosen efficacy and harm outcomes is strongly recommended	14
Participant timeline	#13	Time schedule of enrolment, interventions (including any run-ins and washouts), assessments, and visits for participants. A schematic diagram is highly recommended (see Figure)	10
Sample size	#14	Estimated number of participants needed to achieve study objectives and how it was determined, including clinical and statistical assumptions supporting any sample size calculations	6
Recruitment	#15	Strategies for achieving adequate participant enrolment to reach target sample size	12
**Methods: Assignment of interventions (for controlled trials)**			
Allocation: sequence generation	#16a	Method of generating the allocation sequence (eg, computer-generated random numbers), and list of any factors for stratification. To reduce predictability of a random sequence, details of any planned restriction (eg, blocking) should be provided in a separate document that is unavailable to those who enrol participants or assign interventions	N/A

For peer review only

Allocation concealment mechanism	#16b	Mechanism of implementing the allocation sequence (eg, central telephone; sequentially numbered, opaque, sealed envelopes), describing any steps to conceal the sequence until interventions are assigned	N/A
Allocation: implementation	#16c	Who will generate the allocation sequence, who will enrol participants, and who will assign participants to interventions	N/A
Blinding (masking)	#17a	Who will be blinded after assignment to interventions (eg, trial participants, care providers, outcome assessors, data analysts), and how	N/A
Blinding (masking): emergency unblinding	#17b	If blinded, circumstances under which unblinding is permissible, and procedure for revealing a participant's allocated intervention during the trial	N/A
**Methods: Data collection, management, and analysis**			
Data collection plan	#18a	Plans for assessment and collection of outcome, baseline, and other trial data, including any related processes to promote data quality (eg, duplicate measurements, training of assessors) and a description of study instruments (eg, questionnaires, laboratory tests) along with their reliability and validity, if known. Reference to where data collection forms can be found, if not in the protocol	7
Data collection plan: retention	#18b	Plans to promote participant retention and complete follow-up, including list of any outcome data to be collected for participants who discontinue or deviate from intervention protocols	N/A
Data management	#19	Plans for data entry, coding, security, and storage, including any related processes to promote data quality (eg, double data entry; range checks for data values). Reference to where details of data management procedures can be found, if not in the protocol	N/A
Statistics: outcomes	#20a	Statistical methods for analysing primary and secondary outcomes. Reference to where other details of the statistical analysis plan can be found, if not in the protocol	13-14
Statistics: additional analyses	#20b	Methods for any additional analyses (eg, subgroup and adjusted analyses)	N/A

For peer review only

Statistics: analysis population and missing data	#20c	Definition of analysis population relating to protocol non-adherence (eg, as randomised analysis), and any statistical methods to handle missing data (eg, multiple imputation)	13-14
**Methods: Monitoring**			
Data monitoring: formal committee	#21a	Composition of data monitoring committee (DMC); summary of its role and reporting structure; statement of whether it is independent from the sponsor and competing interests; and reference to where further details about its charter can be found, if not in the protocol. Alternatively, an explanation of why a DMC is not needed	N/A
Data monitoring: interim analysis	#21b	Description of any interim analyses and stopping guidelines, including who will have access to these interim results and make the final decision to terminate the trial	N/A
Harms	#22	Plans for collecting, assessing, reporting, and managing solicited and spontaneously reported adverse events and other unintended effects of trial interventions or trial conduct	N/A
Auditing	#23	Frequency and procedures for auditing trial conduct, if any, and whether the process will be independent from investigators and the sponsor	N/A
**Ethics and dissemination**			
Research ethics approval	#24	Plans for seeking research ethics committee / institutional review board (REC / IRB) approval	1
Protocol amendments	#25	Plans for communicating important protocol modifications (eg, changes to eligibility criteria, outcomes, analyses) to relevant parties (eg, investigators, REC / IRBs, trial participants, trial registries, journals, regulators)	N/A
Consent or assent	#26a	Who will obtain informed consent or assent from potential trial participants or authorised surrogates, and how (see Item 32)	2
Consent or assent: ancillary studies	#26b	Additional consent provisions for collection and use of participant data and biological specimens in ancillary studies, if applicable	2
Confidentiality	#27	How personal information about potential and enrolled participants will be collected, shared, and maintained in order to protect confidentiality before, during, and after the trial	N/A

For peer review only

Declaration of interests	#28	Financial and other competing interests for principal investigators for the overall trial and each study site	19
Data access	#29	Statement of who will have access to the final trial dataset, and disclosure of contractual agreements that limit such access for investigators	N/A
Ancillary and post trial care	#30	Provisions, if any, for ancillary and post-trial care, and for compensation to those who suffer harm from trial participation	N/A
Dissemination policy: trial results	#31a	Plans for investigators and sponsor to communicate trial results to participants, healthcare professionals, the public, and other relevant groups (eg, via publication, reporting in results databases, or other data sharing arrangements), including any publication restrictions	2
Dissemination policy: authorship	#31b	Authorship eligibility guidelines and any intended use of professional writers	N/A
Dissemination policy: reproducible research	#31c	Plans, if any, for granting public access to the full protocol, participant-level dataset, and statistical code	N/A
**Appendices**			
Informed consent materials	#32	Model consent form and other related documentation given to participants and authorised surrogates	N/A
Biological specimens	#33	Plans for collection, laboratory evaluation, and storage of biological specimens for genetic or molecular analysis in the current trial and for future use in ancillary studies, if applicable	7

The SPIRIT Explanation and Elaboration paper is distributed under the terms of the Creative Commons Attribution License CC-BY-NC. This checklist was completed on 04. March 2021 using https://www.goodreports.org/, a tool made by the EQUATOR Network in collaboration with Penelope.ai

For peer review only

The SHAPER-PND trial: clinical effectiveness protocol of a community singing intervention for postnatal depression

Journal:	*BMJ Open*
Manuscript ID	bmjopen-2021-052133.R1
Article Type:	Protocol
Date Submitted by the Author:	15-Sep-2021
Complete List of Authors:	Estevao, Carolina; King's College London Institute of Psychiatry Psychology and Neuroscience, Department of Psychological Medicine<br>Bind, Rebecca; King’s College London, Department of Psychological Medicine<br>Fancourt, Daisy; University College London, Department of Behavioural Science and Health<br>Sawyer, Kristi; King’s College London, Department of Psychological Medicine<br>Dazzan, Paola; King's College London<br>Sevdalis, Nick; King's College London, Centre Implementation Science<br>Woods, Anthony; King’s College London, Department of Psychological Medicine<br>Crane, Nikki; King's College London<br>Rebecchini, Lavinia; King’s College London, Department of Psychological Medicine<br>Hazelgrove, Katie; King’s College London, Department of Psychological Medicine<br>Manoharan, Manonmani; South London and Maudsley NHS Foundation Trust<br>Burton, Alexandra; University College London, Department of Behavioural Science and Health<br>Dye, Hannah; Breathe Arts Health Research<br>Osborn, Tim; Breathe Arts Health Research<br>Davis, Rachel; King's College London, Centre for Implementation Science<br>Soukup, Tayana ; King's College London, Centre for Implementation Science<br>Arias de la Torre, Jorge; King's College London, Centre for Implementation Science; CIBER<br>Bakolis, Ioannis; King's College London, Centre for Implementation Science; King's College London, Department of Biostatistics and Health Informatics<br>Healey, Andy; King's College London, Health Service and Population Research Department<br>Perkins, Rosie; Royal College of Music, Centre for Performance Science; Imperial College London, Faculty of Medicine<br>Pariante, Carmine; King's College London, Psychological Medicine
**Primary Subject Heading**:	Mental health
Secondary Subject Heading:	Qualitative research, Research methods

Keywords:	MENTAL HEALTH, PSYCHIATRY, Depression & mood disorders < PSYCHIATRY, IMMUNOLOGY

I, the Submitting Author has the right to grant and does grant on behalf of all authors of the Work (as defined in the below author licence), an exclusive licence and/or a non-exclusive licence for contributions from authors who are: i) UK Crown employees; ii) where BMJ has agreed a CC-BY licence shall apply, and/or iii) in accordance with the terms applicable for US Federal Government officers or employees acting as part of their official duties; on a worldwide, perpetual, irrevocable, royalty-free basis to BMJ Publishing Group Ltd ("BMJ") its licensees and where the relevant Journal is co-owned by BMJ to the co-owners of the Journal, to publish the Work in this journal and any other BMJ products and to exploit all rights, as set out in our licence.

The Submitting Author accepts and understands that any supply made under these terms is made by BMJ to the Submitting Author unless you are acting as an employee on behalf of your employer or a postgraduate student of an affiliated institution which is paying any applicable article publishing charge ("APC") for Open Access articles. Where the Submitting Author wishes to make the Work available on an Open Access basis (and intends to pay the relevant APC), the terms of reuse of such Open Access shall be governed by a Creative Commons licence – details of these licences and which Creative Commons licence will apply to this Work are set out in our licence referred to above.

Other than as permitted in any relevant BMJ Author's Self Archiving Policies, I confirm this Work has not been accepted for publication elsewhere, is not being considered for publication elsewhere and does not duplicate material already published. I confirm all authors consent to publication of this Work and authorise the granting of this licence.

For peer review only

BMJ OPEN

Title: The SHAPER-PND trial: clinical effectiveness protocol of a community singing intervention for postnatal depression

Authors: Carolina Estevao[1], Rebecca Bind[1], Daisy Fancourt[2], Kristi Sawyer[1], Paola Dazzan[1], Nick Sevdalis[3], Anthony Woods[1], Nikki Crane[4], Lavinia Rebecchini[1], Katie Hazelgrove[1], Manonmani Manoharan[5], Alexandra Burton[2], Hannah Dye[6], Tim Osborn[6], Rachel Davis[3], Tayana Soukup[3], Jorge Arias De La Torre[3,7], Ioannis Bakolis[3,8], Andy Healey[9], Rosie Perkins[10,11], Carmine M. Pariante[1]

[1]Department of Psychological Medicine, Institute of Psychiatry, Psychology and Neuroscience, King's College London, 5 Cutcombe Rd, Brixton, London SE5 9RT, United Kingdom

[2]Department of Behavioural Science and Health, University College London, Gower Street, London, WC1E 6BT, United Kingdom

[3]Centre for Implementation Science, Health Service and Population Research Department, Institute of Psychiatry, Psychology & Neuroscience, 16 De Crespigny Park, Camberwell, London SE5 8AB, United Kingdom

[4]Culture team, King's College London, Somerset House East Wing, Strand WC2R 2LS, United Kingdom

[5]South London and Maudsley NHS Foundation Trust, Denmark Hill, London SE5 8AZ, United Kingdom

[6]Breathe Arts Health Research, The Clarence Centre, 6 St George's Circus, London SE1 6FE, United Kingdom

[7]CIBER Epidemiology and Public Health (CIBERESP), Madrid, Spain

[8]Department of Biostatistics and Health Informatics, Institute of Psychiatry, Psychology & Neuroscience, 16 De Crespigny Park, Camberwell, London SE5 8AB, United Kingdom

[9]King's Health Economics, Health Service and Population Research Department, Institute of Psychiatry, Psychology & Neuroscience, 16 De Crespigny Park, Camberwell, London SE5 8AB, United Kingdom

[10]Centre for Performance Science, Royal College of Music, London, United Kingdom

[11]Faculty of Medicine, Imperial College London, London, United Kingdom

For peer review only

Corresponding author: Carolina Estevao, carolina.estevao@kcl.ac.uk

Keywords: arts intervention, randomised controlled trial, postnatal depression, mental health, singing

Word count: 3,973 words

Abstract

Introduction: Postnatal depression (PND) affects approximately 13% of new mothers. Community-based activities are sought after by many mothers, especially mothers that prefer not to access pharmacological or psychological interventions. Singing has shown positive effects in maternal mood and mother-child bonding. The Scaling-Up Health-Arts Programmes: Implementation and Effectiveness Research – Postnatal Depression (SHAPER-PND) study will analyse the clinical and implementation effectiveness of 10-week singing sessions for PND in new mothers. This protocol paper will focus on the clinical effectiveness of this trial.

Methods and analysis: A total of 400 mothers with PND (with a score of at least 10 on the Edinburgh Postnatal Depression Scale) and their babies will be recruited for this hybrid type II randomised controlled trial The intervention group will attend 10 weekly singing sessions held at community venues or online, facilitated by the arts organisation, Breathe Arts Health Research (Breathe). A control group will be encouraged to attend non-singing sessions in the community or online for 10 weeks. A package of assessments will be collected from participants for clinical, mechanistic and implementation outcomes, at different stages of the trial. Clinical assessments will include questionnaires and interviews for demographics, mental health, and social measures, together with biological samples for measurement of stress markers; the study visits are at baseline, week 6 (mid-trial) and week 10 (end of trial), with follow ups at weeks 20 and 36. Multiple Imputation will be used to deal with possible missing data and multivariable models will be fitted to assess differences between groups in the outcomes of the study.

Ethics and dissemination: Ethical approval has been granted by the London - West London & GTAC Research Ethics Committee, REC reference: 20/PR/0813.

Samples will be handled according to HTA regulations. All data will be handled in line with GDPR and other relevant regulations.

Informed consent will be collected from all research participants and stakeholders involved in the study.

For peer review only - http://bmjopen.bmj.com/site/about/guidelines.xhtml

Findings will be published in peer-reviewed journals and disseminated at national and international meetings. Participants will be made aware of the results of the study through a newsletter.

Article Summary

Strengths and limitations of this study

- Largest cohort of participants in a study examining singing in postnatal depression;
- Largest cohort studying biological measures in relation to an arts intervention for postnatal depression;
- Largest study to analyse the effect of an arts intervention on the mother-infant interaction;
- The state-of-the-art design will result in a unique package of clinical and implementation effectiveness on this intervention;
- There may be inconsistency throughout an intervention period if in-person sessions are switched online due to COVID-19 social distancing restrictions.

Introduction

Postnatal depression

Postnatal depression (PND) affects approximately 13% of new mothers, with symptoms including low mood, fatigue, anhedonia, insomnia and irritability [1,2]. Although pharmacological treatment has yielded positive results, these are hampered by low uptake and adherence, and while mother-infant psychotherapy has produced mixed results, poses similar challenges, including short-lived improvements [2–5].

Prior studies have found that PND carries implications for not only maternal health and wellbeing, but also for the mother-infant relationship [6]. A common finding is that, while interacting with their babies, depressed mothers spend more time in negative, withdrawn states [7,8] and exhibit reduced sensitivity [9,10], and in turn, their infants mirror this behaviour by disengaging and adopting a passive behavioural style [7]. Moreover, depressed mothers are more likely to report reduced feelings of attachment toward their infant [11] and are less likely to mentalize appropriately with their newborn [12]. Maternal sensitivity is highly predictive of infant secure attachment [13], which has long been linked to optimal developmental outcomes including mental health [14]; thus, intervention early-on to ameliorate the mother-infant interaction is warranted in order to protect offspring future development. However, while home-based interventions have been successful in improving PND, they show limited efficacy

For peer review only

in helping the relationship itself [15]. Indeed, a recent combined psychoeducation and play intervention in a community setting was found to decrease dysfunctional parenting behaviours, but the intervention proved more effective in PND symptom reduction than in interaction improvement [16]; and a very small case study found that mother-infant painting sessions were successful in improving both the relationship and PND symptoms, though the sample size was only four dyads [17].

Despite these challenges in finding optimal treatment options, which can both alleviate maternal symptoms of PND and strengthen the mother-infant relationship, many mothers continue to engage in community group activities, such as mother-infant play groups, as they are found to be effective at relaxing mothers, providing good sources of social interaction, decreasing the monotony of each day, and providing a sense of personal fulfilment for mothers [18]. Specifically, there is a growing body of evidence demonstrating the effects of community group singing on mental health [19,20] (see also the Melodies for Mums programme, described below). Singing to newborns is practised around the world, and research has demonstrated valuable benefits such as improving mother-infant interaction and reducing infant distress [21–23]. Listening to music during pregnancy is also associated with greater maternal wellbeing and reduced symptoms of PND in the first 3 months post-birth, and, additionally, daily singing to babies is associated with fewer symptoms of PND and increased maternal wellbeing, self-esteem and perceived mother-infant bond [24]. Consequently, there is a strong theoretical background for why singing could support mothers with PND. It is thought that postnatally depressed women may have difficulty with adherence to dyadic interventions given the nature and challenge of their symptoms [25]; however, no prior studies have assessed whether a community singing-based intervention may be able to both improve the quality of the developing relationship, as well as present as a more engaging intervention that mothers are likelier to complete.

Biological underpinning of singing sessions for postnatal depression

Three biological pathways are suggested to underlie the benefits of music engagement on mood: the glucocorticoid-modulated stress system, the inflammatory system, and the oxytocin system, which is also associated with maternal attachment and bonding. Of these, the hypothalamic-pituitary-adrenal (HPA) axis, of which the hormone cortisol is a main measure, is the most well-studied. Studies in various populations have given evidence for a reduction in salivary cortisol after a singing session [26-29], pointing to a consistent stress-reducing effect of singing. Interestingly, one of these studies also showed that singing sessions produced increases in the levels of cytokines such as IL-(interleukin-)17, IL-2, IL-4 and TNFα, which correlated with the observed reduction in cortisol [27]. The evidence surrounding the oxytocin

For peer review only

system and music participation is more conflicted. Oxytocin is generally shown to increase as a result of singing [30,31], or even listening to music [32]. However, more recent studies have demonstrated a decrease in salivary oxytocin concentrations in certain circumstances, for example after choir singing, but not solo singing [28]. In addition, one study showed a decrease in oxytocin after singing sessions, which was associated with the increase in cytokines and reduction in cortisol, as described above [27]. This was proposed to be indicative of a general dampening of all stress-related signalling, in which oxytocin also plays a part, leading to an overall improved sense of wellbeing.

Studies investigating the interplay of these three biological systems in relation to musical engagement during the perinatal period are sparse. Indeed, only one study has investigated the biological effects of maternal singing groups [33], in which pregnant women were randomised to either music intervention, singing intervention, or control group. This study found a decrease in cortisol in both intervention groups, but that the effect was stronger in the singing group. Additionally, both musical intervention groups showed an increase in salivary oxytocin post-session. This is of particular interest given oxytocin's involvement in the mother-infant relationship [34], which may be strengthened in cases of mother-child biobehavioural synchrony, in which maternal and infant hormone levels covary [35].

The "Melodies for Mums" intervention

Melodies for Mums (M4M) is an intervention that was developed and tested as part of a collaboration between the Royal College of Music, Imperial College London and University College London (UK). M4M was tested in a three-arm randomised controlled trial (RCT) involving 134 mothers with PND (with an Edinburgh Postnatal Depression Scale (EPDS) score ≥ 10 at baseline, indicating depression), and compared the efficacy of community singing with a comparison group (10 weeks of creative play classes for dyads) and a wait-list control group (10 weeks of care-as-usual). This pilot study found that mothers with moderate-severe symptoms of PND, defined as an EPDS score of ≥13, who participated in the programme with their baby, had a significantly faster improvement in symptoms than mothers in usual care [36].

Following the pilot study, M4M was taken-on as a service by Breathe Arts Health Research (Breathe) across the boroughs of Lambeth and Southwark, in South London. However, there has since been no further research on the programme; thus, its effectiveness on a larger scale remains to be evaluated, as do the acceptability, appropriateness and feasibility of its delivery on a larger-scale, and its potential to be adopted and sustained as a cost-effective, beneficial service for mothers within national health services. In addition, there is no evidence on the effects of M4M on mother-infant interaction, and a detailed mechanistic investigation of the

For peer review only - http://bmjopen.bmj.com/site/about/guidelines.xhtml

biological underpinning of any therapeutic effects is also missing. This paper will focus specifically on the protocol for a Wellcome-Trust-funded study aimed to deliver the clinical effectiveness and the biomarkers evaluation of M4M, the “Scaling-Up Health-Arts Programmes: Implementation and Effectiveness Research – Postnatal Depression” (SHAPER-PND) study. Information on the implementation effectiveness of M4M is discussed in a separate detailed protocol.

Methods and analysis

Aims

1) To determine the clinical effectiveness of the intervention in a larger sample size than previous studies (n= 400 women and their babies). This will allow us to ascertain whether the initial findings from the pilot study are replicable on a larger scale. To understand whether singing is an efficacious treatment for PND, including on mother-infant interaction, various psychological, social, and qualitative measures will be collected from women and their infants before, during, and after the intervention period.

2) To ascertain the biological mechanisms by which postnatal singing groups putatively improve maternal mood and mother-infant relationships. This will further the current understanding of the positive effects of the intervention and provide objectively measurable outcomes. Maternal and infant samples will be collected to measure cortisol and oxytocin, plus maternal inflammatory markers.

Objectives and outcomes

To address our aims, we have the following objectives and outcomes. As this protocol paper is focusing only on the clinical effectiveness of the trial, the objectives and outcomes of the implementation component of the trial have been reported in a separate manuscript.

Primary objective

1. Does community singing improve symptoms of PND?

Participants will complete the EPDS prior to the intervention (baseline) to ascertain the severity of their PND. The EPDS will be administered again at various timepoints throughout and following the intervention period (weeks 6, 10, 20 and 36).

Outcome: The primary outcome of this study is to understand whether singing reduces the severity of symptoms of PND on the EPDS between baseline and week 10 (end of intervention), via a reduction in total EPDS score.

For peer review only

Secondary Objectives

2. Does community singing improve further aspects of maternal mental health?

Further measures of mental health will be administered at baseline and again at weeks 6, 10, 20 and 36.. As such, the Beck Depression Inventory (BDI) and the Hamilton Depression Rating Scale (HAM-D) will be administered to assess further traits of depression; the State-Trait Anxiety Inventory (STAI) and Perceived Stress Scale (PSS) will be administered to evaluate levels of anxiety and stress; and the Office for National Statistics Wellbeing Scale (ONS) will be used to gather information on general satisfaction and wellbeing.

Outcome: The outcome is to assess whether singing ameliorates further aspects of mental health in women with PND, including depressive symptoms, anxiety, stress, and wellbeing, between baseline and week 10, via a reduction in score on the SCID-5, BDI, HAM-D, STAI, and PSS, and an increase in ONS score.

3. Does community singing facilitate the mother-infant relationship?

The study will evaluate aspects of the dyad at baseline, and again throughout the intervention. Specifically, mothers will complete the Maternal Postnatal Attachment Scale (MPAS) and the Parental Reflective Functioning Questionnaire (PRFQ) at baseline and weeks 6, 10, 20 and 36, to report on their subjective experience of the relationship; and, furthermore, dyads will be filmed at baseline and weeks 10 and 36 and rated for the quality of the interaction according to the Crittenden Child-Adult Relationship Experimental Index (CCI), also known as the CARE-Index.

Outcome: The outcome is to understand whether singing aids in improving upon aspects of the mother-infant relationship in women with PND, including attachment and ability for mentalization, between baseline and week 10 via an increase in score on the CARE-Index, MPAS, and PRFQ.

4. Does community singing reduce loneliness and improve social support?

Participants will be asked to complete the UCLA Loneliness Scale, the Short General Self-Efficacy Scale (GSE-6), and the Multidimensional Scale of Perceived Social Support (MSPSS) at baseline and again throughout the intervention period at weeks 6, 10, 20 and 36.

Outcome: The outcome is to evaluate whether singing reduces perceived loneliness and improves upon felt social support in women with PND between baseline and week 10, via a reduction in score on the UCLA Loneliness Scale and an increase in score on the GSE-6 and MSPSS.

5. Are there certain biological mechanisms which underpin psychological change?

For peer review only

Biological samples will be taken from mothers and their infants throughout the course of the intervention. The dyad will each provide diurnal saliva samples before and after the 10-week intervention Furthermore, we will collect a saliva sample from the dyad before and after their first, sixth and tenth sessions. Finally, to assess overall cortisol output over the 10-week period, mothers will provide a hair sample at the end of the intervention.

Outcome: The outcome is to assess whether biological mechanisms underpin the psychological change from singing in women with PND, via changes in their diurnal cortisol and overall cortisol output between baseline and week 10, and furthermore, via changes in oxytocin, cortisol, and inflammatory markers from individual music session at weeks 1, 6, and 10.

6. Does community singing affect the lived experience of women with PND?

Focus groups will be conducted with women who have participated in the intervention. More specifically, the focus groups will capture general feedback from sessions and experiences from mothers in the groups.

Outcome: The outcome is to identify how the singing sessions affect the lived experience of mothers with PND and to understand their reported mechanisms of effect through focus groups.

7. How does community singing interact with PND in women with particular risk factors for PND?

Individual or small-group semi-structured interviews with three sub-groups of women self-reporting particular risk factors for PND (traumatic birth, adverse childhood experiences, social isolation/loneliness) will be conducted.

Outcome: The outcome is to evaluate the phenomenology of PND and understand how singing intersects with the specific context of PND among these sub-groups of women.

Data collection plan

The study is anticipated to run from September 2021 to December 2023.

In order to address the objectives within the clinical effectiveness arm of the study, data will be collected according to the data collection timeline in Table 1.

Table 1 Clinical effectiveness data collection timeline.

**Study objective**	**Type of measure**	**Measures**	**Timepoint collected**	**Type of data**

For peer review only

1	Mental health	Edinburgh Postnatal Depression Scale (EPDS)	Baseline, weeks 1, 6, 10, 20 & 36	Quantitative
2	Mental health	Hamilton Depression Rating Scale (HAM-D), Structured Clinical Interview for DSM-IV Disorders (SCID)	Weeks 1 & 10	Quantitative
		Beck Depression Inventory (BDI), State Trait Anxiety Inventory (STAI), Perceived Stress Scale (PSS); Office for National Statistics Wellbeing Scale (ONS)	Weeks 1, 6, 10, 20 & 36	
3	Social	Maternal Postpartum Attachment Scale (MPAS), Parental Reflective Functioning Questionnaire (PRFQ)	Weeks 1, 6, 10, 20 & 36	Quantitative
		Crittenden Child-Adult Relationship (CCI CARE-Index)[1]	Baseline, weeks 10 & 36	
4	Social	UCLA Loneliness Scale, Short General Self-Efficacy Scale (GSE-6), Multidimensional Scale of Perceived Social Support (MSPSS)	Weeks 1, 6, 10, 20 & 36	Quantitative
5	Biological	Diurnal cortisol saliva samples[1]	Baseline & week 10	Quantitative
		Pre- and post-session saliva samples[1]	Weeks 1, 6, 10 (at sessions)	

For peer review only

		Hair samples	Week 10	
6	Qualitative	Focus groups with intervention groups to capture feedback from sessions and experiences from mothers in the groups, focusing on the lived experience of PND	Week 10	Qualitative
7	Qualitative	Semi-structured interviews with a sub sample of the intervention group of women self-reporting particular risk factors for PND (partially informed by demographic questionnaires)	Weeks 10-20, informed by information collected at baseline	Qualitative

[1] Collected from mothers and infants

In addition to the measures collected above, we will also collect comprehensive information on maternal and infant socio-demographics and medical health, maternal history of childhood maltreatment, and current maternal abuse, in order to explore the interaction between notable risk factors and the development of PND.

Trial Design

Scaling-Up Health-Arts Programmes: Implementation and Effectiveness Research (SHAPER) is a multi-disciplinary research programme funded by the Wellcome Trust that is hosted by King's College London (Sponsor) and University College London. The SHAPER-PND study described here is Hybrid Type II Effectiveness-Implementation randomised-controlled trial, where equal focus is placed on the effectiveness of the intervention and the effectiveness of its implementation [37].

Study Flowchart

An overview of the study timelines and procedures has been summarised in the flowchart below (

Figure 1):

For peer review only

For peer review only

Setting

This is a multi-centre trial that will be run in locations across London (primarily in the South London boroughs of Lewisham, Lambeth and Southwark), specifically in children's or community venues or online. These venues will be thoroughly safety- and risk-assessed in light of COVID-19 and adherence to any COVID-secure measures will be closely monitored by Breathe.

In order to enrol a sufficiently large sample of women, there will be 12 blocks of the 10-week singing programme in total: 10 intervention blocks will be for the experimental group, and 2 blocks will be for women in the wait-list control group that wish to take part in the singing sessions. The 2 wait-list blocks will be offered singing sessions after a woman has completed the first 10 weeks of the study and no data will be collected regarding these singing sessions, apart from the control follow-up data that this group is expected to provide.

In the event that one or several women need to self-isolate due to confirmed or suspected exposure to COVID-19, the sessions will be delivered to her/them online via Zoom. This will allow for the sessions to still be conducted in person with the remaining women.

In the event of the artist or the Breathe officer having to self-isolate, a replacement Breathe-trained artist will be ready to step-in and deliver the sessions. The same will apply to Breathe staff; there will be another staff member available to be present at the sessions.

Participants

Study participants will be new mothers exhibiting symptoms of PND, who meet criteria for a current major depressive episode, and have babies aged 0-9 months.

Inclusion Criteria: Women aged 18 or older; satisfactory understanding of English; women with infants aged 0-9 months; women with PND, who score ≥10 on the EPDS. Women will require access to an internet-connected device (mobile phone, tablet, computer or laptop) to allow completion of assessments and participation in the singing sessions in the event they go online.

Exclusion Criteria: The participant may not enter the study if her baby is outside of the age-range specified; if she is unable to give informed consent.

Number of participants and power analysis

The pilot RCT that informed this study showed that depressive symptoms (EPDS scores) improved in both singing and control women by the end of treatment (week 10), but the singing group had a numerically larger improvement (-5.2 (SD=2.8)) than the care-as-usual group (-

For peer review only

4.25 (SD=3.2), effect size=0.32)) [36]. To have power of 80% (at $p<0.05$, two-tailed) to detect the same effect size difference, randomising subjects 2:1, 232 subjects will be required in the intervention and 116 in the control group (total= 348). In total, 400 mothers and their babies will be recruited to compensate for a 12% drop-out rate.

Participants will be recruited in blocks of 40-50 women and their babies, screened and assessed, prior to a 2:1 allocation randomisation (dyads will be allocated the same group). This process will be repeated 12 times, aiming to reach 400 dyads.

Recruitment process

The recruitment period will last two years (2021-2022). All potential participants will initially be directed to a pre-screening online form that includes an EPDS. If the EPDS score is < 10, a participant will be notified that she is not currently eligible to participate and will be signposted to other support services within the community (e.g., talking therapies, mother-baby groups, baby activity groups, etc). If a woman's score is ≥ 10, she will be notified that she is potentially eligible and will proceed straight to the second phase of screening through a home (or Zoom) visit. The participant information sheet (PIS) for the trial will be sent once the baseline assessment is booked.

When a new round of 10 classes becomes available, researchers will arrange assessments with potential participants to undertake the full screening interview against the inclusion/exclusion criteria. If the participant is found non-eligible, she will be signposted to other support services within the community.

The singing group

M4M is a 10-week intervention for mothers with PND. The programme will be delivered to groups of 8–12 mothers in weekly sessions lasting one hour. Mothers will start a block of 10-week classes together and continue with the same group and leader for the duration of the course. Classes are free to attend and will take place in Children's Centres or other community venues (or online via Zoom). Mothers will attend with their babies and will be invited to sit in a socially distanced circle on the floor surrounded by soft play cushions and mats. Classes will start with welcome songs, introducing the babies and mothers to one another, and then involve a range of singing and music activities. Mothers will be required to respect social distancing guidelines and keep their babies within their reach to avoid physical contact with other mothers or their babies.

Some songs will be accompanied by maracas, drums, hand chimes and other simple instruments that the mothers and babies can play together. Instruments such as guitar and ukulele will also be used for a small number of songs. Instruments will not be shared between

For peer review only

the participants and will be disinfected before and after the singing sessions with a COVID-19 validated disinfectant.

Mothers will also work to write some of their own songs over the weeks, developing lyrics together about their babies or experiences of motherhood and creating simple melodies. Recordings of the group singing the songs together will be made and uploaded to private online platforms or onto CDs for the mothers to listen to at home. Classes will be led by professional workshop leaders trained by Breathe, with support of assistants.

The control group

The control group will be an ‘active’ control. During the first 10 weeks (during the study period), mothers in the control group will receive details of other non-music classes available to them in the community (either in-person or online, depending on the programs available at the time and government guidelines) and will receive the same schedule of texts and phone calls to encourage them to join these activities. They will still be seen by the researchers to collect clinical measures and biological samples (including the pre-post saliva samples) and to monitor engagement in other activities. Following the first 10 weeks, the mothers in the control group will be offered a place on the singing programme, but these data will not be part of the study, and they will not join groups with other women who are in the study.

Patient and public involvement

Participants from previous sessions delivered by Breathe in the community have provided valuable feedback and insights into the sessions’ delivery, content and schedule. These were gathered through focus groups and workshops where mothers and others involved in the singing sessions were able to share their experience of the sessions. This body of work allowed the refinement of the sessions for the intervention to be more suitable for the population.

A previous RCT was designed and delivered by two of this study’s co-authors (DF, RP), along with Breathe [36]. The RCT analysed the clinical efficacy of the intervention on a smaller scale and explored the experiences of participants receiving and delivering the intervention, which ultimately informed the design of this SHAPER-PND study.

Data Analyses

Quantitative analyses

The main statistical analyses will be carried out by the trial statistician who will be blind to the group randomisation until the main analyses are complete. The analyses outlined in this

For peer review only

strategy will be primarily based on intention to treat. A per-protocol analysis will also be conducted in which the primary outcome will also be compared between groups, removing data of participants who:

1. Do not complete their intervention as defined above. 2. Miss over 50% of the singing or control sessions. 3. Are later found to have an exclusion criterion that was missed.

The first stage of analyses will be a descriptive model of the data to assess their completeness. Initially, a complete case analysis was performed under a missing at random assumption (MAR) where explanatory variables could predict the missing values in the respective outcome variable. These models drop any participant who does not provide outcome data at the follow up time point.

Mother-level baseline variables will be described both overall and by randomised groups. Patterns of missing data will be described, if any. predictors of missingness will be also assessed. The primary outcome will be analysed using linear mixed models to model the change in total score of EPDS at 6-, 10-, 20- and 36-weeks post randomisation. A three-level hierarchical model will be employed with all-time points included as repeated measures in the model (6-, 10-, 20- and 36-weeks post randomisation) adjusting for baseline EPDS to improve power and take into account clustering of the observation at mother's level. Associations between post randomisation variables and missingness will be dealt with by multiple imputation by chained equations, again under the MAR assumption. Departures from this assumption will be assessed with a sensitivity analysis. All baseline characteristics as well as the outcomes will be included in the MI models. Secondary outcomes (e.g., PSS, HAM-D) will be assessed with a similar methodology for the primary outcomes, using generalised linear mixed models depending on the type of outcome (normal, binary and count). The questionnaires to be used have validated methods of scoring, and the scores will be analysed as described. All analyses will be conducted in STATA V.15.1.

For peer review only

Qualitative analyses

Data from the focus groups will be analysed using Thematic Analysis [38], to understand the lived experience of PND and how this relates to experiences in the singing group. Data from the individual/small-group interviews with sub-groups of women self-reporting particular risk factors for PND (traumatic birth, adverse childhood experiences, and social isolation/loneliness) will be analysed using Interpretative Phenomenological Analysis (IPA) [39] to focus in-depth on how singing intersects with the specific context of PND among women in these sub-groups.

Ethics and dissemination

Ethical approval has been granted by the London - West London & GTAC Research Ethics Committee, REC reference: 20/PR/0813.

Samples will be handled according to HTA regulations. All data will be handled in line with GDPR and other relevant regulations.

Informed consent will be collected from all research participants and stakeholders involved in the study.

Findings will be published in peer-reviewed journals and disseminated at national and international meetings. Participants will be made aware of the results of the study through a newsletter.

Contributorship statement

All authors listed have contributed to the conception and design of the protocol and this manuscript. All authors have been involved in the drafting of the manuscript and have individually approved the version of the work published.
Specifically, the contribution of each author falls within the following CRediT categories:
CE, RB, KS, PD, NS, LR, KH, AB, RD, TS, JAT, IB and AH : conceptualization, methodology and project administration.

DF, RP, CP: conceptualisation, methodology, supervision and funding acquisition.

NC, AW: conceptualisation, project administration and funding acquisition.

MM, HD and TO: conceptualisation, project administration.

Competing interests

NS is the director of the London Safety and Training Solutions Ltd, which offers training in patient safety, implementation solutions and human factors to healthcare organisations.

DF is a non-executive board director for Breathe Arts Health Research but she receives no financial compensation for her involvement.

TS received funding for training cancer multidisciplinary teams in the assessment and quality improvement methods in the United Kingdom. TS serves as a consultant to F. Hoffmann-La Roche Diagnostics providing advisory research services in relation to innovations for cancer multidisciplinary teams and their care planning meetings in the United States of America.

For peer review only

CMP has received research and consultation funding from Boehringer Ingelheim and Johnson & Johnson for research on depression and inflammation, and by a Wellcome Trust strategy award to the Neuroimmunology of Mood Disorders and Alzheimer's Disease (NIMA) Consortium (104025), which is also funded by Janssen, GlaxoSmithKline, Lundbeck and Pfizer; the work presented in this paper is unrelated to this funding.

The other authors have no conflicts of interest to declare.

Funding Statement

This trial is part of the SHAPER programme, a Scaling-up Health-Arts Programme to scale up arts interventions. This programme is funded by the Wellcome Trust (award reference 219425/Z/19/Z). This work is additionally supported by the National Institute for Health Research (NIHR) Biomedical Research Centre at South London and Maudsley NHS Foundation Trust and King's College London and by a NIHR Senior Investigator to CMP.

NS, IB and AH' research is supported by the National Institute for Health Research (NIHR) Applied Research Collaboration (ARC) South London at King's College Hospital NHS Foundation Trust. NS and AH are members of King's Improvement Science, which offers co-funding to the NIHR ARC South London and comprises a specialist team of improvement scientists and senior researchers based at King's College London. Its work is funded by King's Health Partners (Guy's and St Thomas' NHS Foundation Trust, King's College Hospital NHS Foundation Trust, King's College London and South London and Maudsley NHS Foundation Trust), Guy's and St Thomas' Charity and the Maudsley Charity. The views expressed in this publication are those of the authors and not necessarily those of the Wellcome Trust, the NIHR, or the Department of Health and Social Care.

RD is supported by the National Institute for Health Research (NIHR) Applied Research Collaboration: South London, at King's College Hospital NHS Foundation Trust. The views expressed in this publication are those of the author(s) and not necessarily those of the NHS, NIHR or the Department of Health and Social Care.

Data sharing statement: Not applicable, this manuscript does not contain any data.

References

1 Boath E, Henshaw C. The treatment of postnatal depression: A comprehensive literature review. *J Reprod Infant Psychol* 2001;**19**:215–48. doi:10.1080/02646830120073224

For peer review only

2 Morrell C, Warner R, Slade P, *et al.* Psychological interventions for postnatal depression: cluster randomised trial and economic evaluation. The PoNDER trial. *Health Technol Assess* 2009;**13**. doi:10.3310/hta13300

3 Bledsoe SE, Grote NK. Treating Depression During Pregnancy and the Postpartum: A Preliminary Meta-Analysis. *Res Soc Work Pract* 2006;**16**:109–20. doi:10.1177/1049731505282202

4 Cooper PJ, Murray L, Wilson A, *et al.* Controlled trial of the short- and long-term effect of psychological treatment of post-partum depression 1. Impact on maternal mood. *Br J Psychiatry* 2003;**182**:412–9. doi:10.1192/bjp.182.5.412

5 Milgrom J, Negri LM, Gemmill AW, *et al.* A randomized controlled trial of psychological interventions for postnatal depression. *Br J Clin Psychol Br Psychol Soc* 2005;**44**:529–42. doi:10.1348/014466505X34200

6 Murray L, Fearon P, Cooper P. Postnatal depression, mother-infant interactions, and child development: Prospects for screening and treatment. In: *Identifying perinatal depression and anxiety: Evidence-based practice in screening, psychosocial assessment, and management.* Wiley-Blackwell 2015. 139–64.

7 Field T, Healy BT, Goldstein S, *et al.* Behavior-state matching and synchrony in mother-infant interactions of nondepressed versus depressed dyads. *Dev Psychol* 1990;**26**:7–14. doi:10.1037/0012-1649.26.1.7

8 Field T. Maternal Depression Effects on Infants and Early Interventions. *Prev Med* 1998;**27**:200–3. doi:10.1006/pmed.1998.0293

9 Field T. Postpartum depression effects on early interactions, parenting, and safety practices: A review. *Infant Behav Dev* 2010;**33**:1–6. doi:10.1016/j.infbeh.2009.10.005

10 Murray L, Fiori-Cowley A, Hooper R, *et al.* The impact of postnatal depression and associated adversity on early mother-infant interactions and later infant outcome. *Child Dev* 1996;**67**:2512–26.

11 Moehler E, Brunner R, Wiebel A, *et al.* Maternal depressive symptoms in the postnatal period are associated with long-term impairment of mother–child bonding. *Arch Womens Ment Health* 2006;**9**:273–8. doi:10.1007/s00737-006-0149-5

12 Pawlby S, Fernyhough C, Meins E, *et al.* Mind-mindedness and maternal responsiveness in infant–mother interactions in mothers with severe mental illness. *Psychol Med* 2010;**40**:1861–9. doi:10.1017/S0033291709992340

13 Campbell SB, Brownell CA, Hungerford A, *et al.* The course of maternal depressive symptoms and maternal sensitivity as predictors of attachment security at 36 months. *Dev Psychopathol* 2004;**16**. doi:10.1017/S0954579404044499

14 DeKlyen M, Greenberg M. Attachment and psychopathology in childhood. In: *Handbook of attachment: Theory, research, and clinical applications*. The Guilford Press 2008. 637–65.

15 Gelfand DM, Teti DM, Seiner SA, *et al.* Helping mothers fight depression: Evaluation of a home-based intervention program for depressed mothers and their infants. *J Clin Child Psychol* 1996;**25**:406–22. doi:10.1207/s15374424jccp2504_6

For peer review only

16 Ericksen J, Loughlin E, Holt C, *et al.* A therapeutic playgroup for depressed mothers and their infants: feasibility study and pilot randomized trial of community hugs. *Infant Ment Health J* 2018;**39**:396–409. doi:10.1002/imhj.21723

17 Arroyo C, Fowler N. Before and after: A mother and infant painting group. *Int J Art Ther* 2013;**18**:98–112. doi:10.1080/17454832.2013.844183

18 Feeley N, Bell L, Hayton B, *et al.* Care for Postpartum Depression: What Do Women and Their Partners Prefer? *Perspect Psychiatr Care* 2016;**52**:120–30. doi:10.1111/ppc.12107

19 Clift S, Morrison I. Group singing fosters mental health and wellbeing: findings from the East Kent “singing for health” network project. *Ment Health Soc Incl* 2011;**15**:88–97. doi:10.1108/20428301111140930

20 Coulton S, Clift S, Skingley A, *et al.* Effectiveness and cost-effectiveness of community singing on mental health-related quality of life of older people: randomised controlled trial. *Br J Psychiatry J Ment Sci* 2015;**207**:250–5. doi:10.1192/bjp.bp.113.129908

21 Mualem O, Klein PS. The communicative characteristics of musical interactions compared with play interactions between mothers and their one-year-old infants. *Early Child Dev Care* 2013;**183**:899–915. doi:10.1080/03004430.2012.688824

22 Nakata T, Trehub SE. Infants’ responsiveness to maternal speech and singing. *Infant Behav Dev* 2004;**27**:455–64. doi:10.1016/j.infbeh.2004.03.002

23 Vlismas W, Malloch S, Burnham D. The effects of music and movement on mother–infant interactions. *Early Child Dev Care* 2013;**183**:1669–88. doi:10.1080/03004430.2012.746968

24 Fancourt D, Perkins R. Associations between singing to babies and symptoms of postnatal depression, wellbeing, self-esteem and mother-infant bond. *Public Health* 2017;**145**:149–52. doi:10.1016/j.puhe.2017.01.016

25 Nylen KJ, Moran TE, Franklin CL, *et al.* Maternal depression: A review of relevant treatment approaches for mothers and infants. *Infant Ment Health J* 2006;**27**:327–43. doi:10.1002/imhj.20095

26 Fancourt D, Aufegger L, Williamon A. Low-stress and high-stress singing have contrasting effects on glucocorticoid response. *Front Psychol* 2015;**6**. doi:10.3389/fpsyg.2015.01242

27 Fancourt D, Williamon A, Carvalho LA, *et al.* Singing modulates mood, stress, cortisol, cytokine and neuropeptide activity in cancer patients and carers. *ecancermedicalscience* 2016;**10**. doi:10.3332/ecancer.2016.631

28 Schladt TM, Nordmann GC, Emilius R, *et al.* Choir versus Solo Singing: Effects on Mood, and Salivary Oxytocin and Cortisol Concentrations. *Front Hum Neurosci* 2017;**11**:430. doi:10.3389/fnhum.2017.00430

29 Grebosz-Haring K, Thun-Hohenstein L. Effects of group singing versus group music listening on hospitalized children and adolescents with mental disorders: A pilot study. *Heliyon* 2018;**4**:e01014. doi:10.1016/j.heliyon.2018.e01014

30 Grape C, Sandgren M, Hansson L-O, *et al.* Does singing promote well-being?: An empirical study of professional and amateur singers during a singing lesson. *Integr Physiol Behav Sci* 2002;**38**:65–74. doi:10.1007/BF02734261

For peer review only

31 Kreutz G. Does singing promote social bonding? *Music Med* 2014;**6**:51–60.

32 Nilsson U. Soothing music can increase oxytocin levels during bed rest after open-heart surgery: a randomised control trial. *J Clin Nurs* 2009;**18**:2153–61. doi:10.1111/j.1365-2702.2008.02718.x

33 Wulff V, Hepp P, Wolf OT, *et al.* The effects of a music and singing intervention during pregnancy on maternal well-being and mother–infant bonding: a randomised, controlled study. *Arch Gynecol Obstet* Published Online First: 10 August 2020. doi:10.1007/s00404-020-05727-8

34 Galbally M, Lewis AJ, van IJzendoorn M, *et al.* The Role of Oxytocin in Mother-Infant Relations: A Systematic Review of Human Studies: *Harv Rev Psychiatry* 2011;**19**:1–14. doi:10.3109/10673229.2011.549771

35 Feldman R. Oxytocin and social affiliation in humans. *Horm Behav* 2012;**61**:380–91. doi:10.1016/j.yhbeh.2012.01.008

36 Fancourt D, Perkins R. Effect of singing interventions on symptoms of postnatal depression: three-arm randomised controlled trial. *Br J Psychiatry* 2018;**212**:119–21. doi:10.1192/bjp.2017.29

37 Curran GM, Bauer M, Mittman B, *et al.* Effectiveness-implementation hybrid designs: combining elements of clinical effectiveness and implementation research to enhance public health impact. *Med Care* 2012;**50**:217–26. doi:10.1097/MLR.0b013e3182408812

38 Smith JA. *Qualitative Psychology: A Practical Guide to Research Methods*. SAGE 2015.

39 Smith JA, Shinebourne P. Interpretative phenomenological analysis. In: *APA handbook of research methods in psychology, Vol 2: Research designs: Quantitative, qualitative, neuropsychological, and biological*. Washington, DC, US: : American Psychological Association 2012. 73–82. doi:10.1037/13620-005

Figure legend:

Figure 1 SHAPER-PND study flowchart.

For peer review only

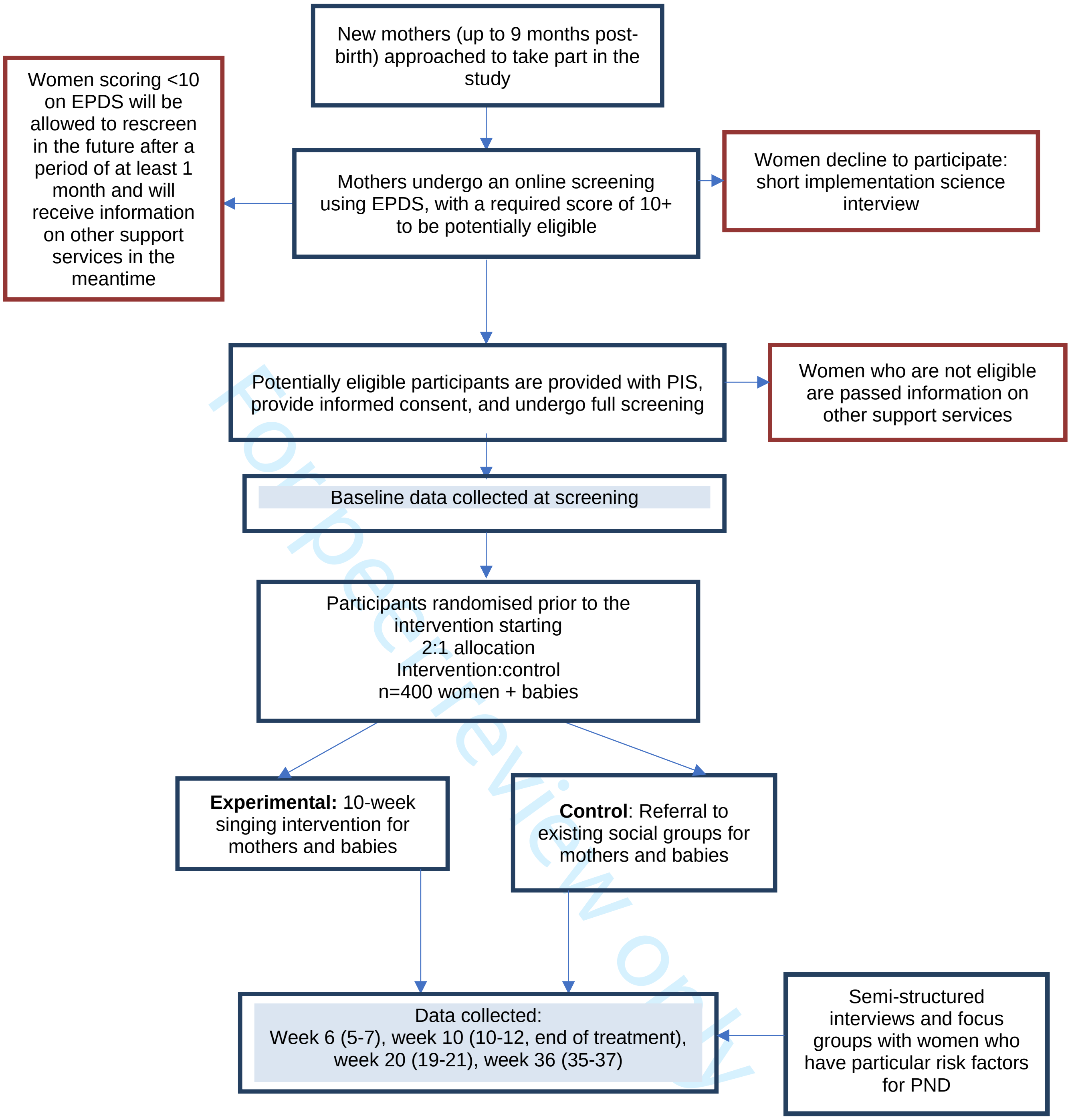
New mothers (up to 9 months post-birth) approached to take part in the study
Women scoring <10 on EPDS will be allowed to rescreen in the future after a period of at least 1 month and will receive information on other support services in the meantime
Mothers undergo an online screening using EPDS, with a required score of 10+ to be potentially eligible
Women decline to participate: short implementation science interview
Potentially eligible participants are provided with PIS, provide informed consent, and undergo full screening
Women who are not eligible are passed information on other support services
Baseline data collected at screening
Participants randomised prior to the intervention starting
2:1 allocation
Intervention:control
n=400 women + babies
Experimental: 10-week singing intervention for mothers and babies
Control: Referral to existing social groups for mothers and babies
Data collected:
Week 6 (5-7), week 10 (10-12, end of treatment), week 20 (19-21), week 36 (35-37)
Semi-structured interviews and focus groups with women who have particular risk factors for PND

Reporting checklist for protocol of a clinical trial.

Based on the SPIRIT guidelines.

Instructions to authors

Complete this checklist by entering the page numbers from your manuscript where readers will find each of the items listed below.

Your article may not currently address all the items on the checklist. Please modify your text to include the missing information. If you are certain that an item does not apply, please write "n/a" and provide a short explanation.

Upload your completed checklist as an extra file when you submit to a journal.

In your methods section, say that you used the SPIRITreporting guidelines, and cite them as:

Chan A-W, Tetzlaff JM, Gøtzsche PC, Altman DG, Mann H, Berlin J, Dickersin K, Hróbjartsson A, Schulz KF, Parulekar WR, Krleža-Jerić K, Laupacis A, Moher D. SPIRIT 2013 Explanation and Elaboration: Guidance for protocols of clinical trials. BMJ. 2013;346:e7586

		Reporting Item	Page Number
**Administrative information**			
Title	#1	Descriptive title identifying the study design, population, interventions, and, if applicable, trial acronym	1
Trial registration	#2a	Trial identifier and registry name. If not yet registered, name of intended registry	1
Trial registration: data set	#2b	All items from the World Health Organization Trial Registration Data Set	1-14
Protocol version	#3	Date and version identifier	1
Funding	#4	Sources and types of financial, material, and other support	19-20
Roles and responsibilities: contributorship	#5a	Names, affiliations, and roles of protocol contributors	1

For peer review only

Roles and responsibilities: sponsor contact information	#5b	Name and contact information for the trial sponsor	9
Roles and responsibilities: sponsor and funder	#5c	Role of study sponsor and funders, if any, in study design; collection, management, analysis, and interpretation of data; writing of the report; and the decision to submit the report for publication, including whether they will have ultimate authority over any of these activities	9
Roles and responsibilities: committees	#5d	Composition, roles, and responsibilities of the coordinating centre, steering committee, endpoint adjudication committee, data management team, and other individuals or groups overseeing the trial, if applicable (see Item 21a for data monitoring committee)	N/A
**Introduction**			
Background and rationale	#6a	Description of research question and justification for undertaking the trial, including summary of relevant studies (published and unpublished) examining benefits and harms for each intervention	3-5
Background and rationale: choice of comparators	#6b	Explanation for choice of comparators	9
Objectives	#7	Specific objectives or hypotheses	6-7
Trial design	#8	Description of trial design including type of trial (eg, parallel group, crossover, factorial, single group), allocation ratio, and framework (eg, superiority, equivalence, non-inferiority, exploratory)	9
**Methods: Participants, interventions, and outcomes**			
Study setting	#9	Description of study settings (eg, community clinic, academic hospital) and list of countries where data will be collected. Reference to where list of study sites can be obtained	11
Eligibility criteria	#10	Inclusion and exclusion criteria for participants. If applicable, eligibility criteria for study centres and individuals who will	12

For peer review only

		perform the interventions (eg, surgeons, psychotherapists)	
Interventions: description	#11a	Interventions for each group with sufficient detail to allow replication, including how and when they will be administered	5
Interventions: modifications	#11b	Criteria for discontinuing or modifying allocated interventions for a given trial participant (eg, drug dose change in response to harms, participant request, or improving / worsening disease)	N/A
Interventions: adherance	#11c	Strategies to improve adherence to intervention protocols, and any procedures for monitoring adherence (eg, drug tablet return; laboratory tests)	N/A
Interventions: concomitant care	#11d	Relevant concomitant care and interventions that are permitted or prohibited during the trial	N/A
Outcomes	#12	Primary, secondary, and other outcomes, including the specific measurement variable (eg, systolic blood pressure), analysis metric (eg, change from baseline, final value, time to event), method of aggregation (eg, median, proportion), and time point for each outcome. Explanation of the clinical relevance of chosen efficacy and harm outcomes is strongly recommended	14
Participant timeline	#13	Time schedule of enrolment, interventions (including any run-ins and washouts), assessments, and visits for participants. A schematic diagram is highly recommended (see Figure)	10
Sample size	#14	Estimated number of participants needed to achieve study objectives and how it was determined, including clinical and statistical assumptions supporting any sample size calculations	6
Recruitment	#15	Strategies for achieving adequate participant enrolment to reach target sample size	12
**Methods: Assignment of interventions (for controlled trials)**			
Allocation: sequence generation	#16a	Method of generating the allocation sequence (eg, computer-generated random numbers), and list of any factors for stratification. To reduce predictability of a random sequence, details of any planned restriction (eg, blocking) should be provided in a separate document that is unavailable to those who enrol participants or assign interventions	N/A

For peer review only

Allocation concealment mechanism	#16b	Mechanism of implementing the allocation sequence (eg, central telephone; sequentially numbered, opaque, sealed envelopes), describing any steps to conceal the sequence until interventions are assigned	N/A
Allocation: implementation	#16c	Who will generate the allocation sequence, who will enrol participants, and who will assign participants to interventions	N/A
Blinding (masking)	#17a	Who will be blinded after assignment to interventions (eg, trial participants, care providers, outcome assessors, data analysts), and how	N/A
Blinding (masking): emergency unblinding	#17b	If blinded, circumstances under which unblinding is permissible, and procedure for revealing a participant's allocated intervention during the trial	N/A
**Methods: Data collection, management, and analysis**			
Data collection plan	#18a	Plans for assessment and collection of outcome, baseline, and other trial data, including any related processes to promote data quality (eg, duplicate measurements, training of assessors) and a description of study instruments (eg, questionnaires, laboratory tests) along with their reliability and validity, if known. Reference to where data collection forms can be found, if not in the protocol	7
Data collection plan: retention	#18b	Plans to promote participant retention and complete follow-up, including list of any outcome data to be collected for participants who discontinue or deviate from intervention protocols	N/A
Data management	#19	Plans for data entry, coding, security, and storage, including any related processes to promote data quality (eg, double data entry; range checks for data values). Reference to where details of data management procedures can be found, if not in the protocol	N/A
Statistics: outcomes	#20a	Statistical methods for analysing primary and secondary outcomes. Reference to where other details of the statistical analysis plan can be found, if not in the protocol	13-14
Statistics: additional analyses	#20b	Methods for any additional analyses (eg, subgroup and adjusted analyses)	N/A

For peer review only

Statistics: analysis population and missing data	#20c	Definition of analysis population relating to protocol non-adherence (eg, as randomised analysis), and any statistical methods to handle missing data (eg, multiple imputation)	13-14
**Methods: Monitoring**			
Data monitoring: formal committee	#21a	Composition of data monitoring committee (DMC); summary of its role and reporting structure; statement of whether it is independent from the sponsor and competing interests; and reference to where further details about its charter can be found, if not in the protocol. Alternatively, an explanation of why a DMC is not needed	N/A
Data monitoring: interim analysis	#21b	Description of any interim analyses and stopping guidelines, including who will have access to these interim results and make the final decision to terminate the trial	N/A
Harms	#22	Plans for collecting, assessing, reporting, and managing solicited and spontaneously reported adverse events and other unintended effects of trial interventions or trial conduct	N/A
Auditing	#23	Frequency and procedures for auditing trial conduct, if any, and whether the process will be independent from investigators and the sponsor	N/A
**Ethics and dissemination**			
Research ethics approval	#24	Plans for seeking research ethics committee / institutional review board (REC / IRB) approval	1
Protocol amendments	#25	Plans for communicating important protocol modifications (eg, changes to eligibility criteria, outcomes, analyses) to relevant parties (eg, investigators, REC / IRBs, trial participants, trial registries, journals, regulators)	N/A
Consent or assent	#26a	Who will obtain informed consent or assent from potential trial participants or authorised surrogates, and how (see Item 32)	2
Consent or assent: ancillary studies	#26b	Additional consent provisions for collection and use of participant data and biological specimens in ancillary studies, if applicable	2
Confidentiality	#27	How personal information about potential and enrolled participants will be collected, shared, and maintained in order to protect confidentiality before, during, and after the trial	N/A

For peer review only

Declaration of interests	#28	Financial and other competing interests for principal investigators for the overall trial and each study site	19
Data access	#29	Statement of who will have access to the final trial dataset, and disclosure of contractual agreements that limit such access for investigators	N/A
Ancillary and post trial care	#30	Provisions, if any, for ancillary and post-trial care, and for compensation to those who suffer harm from trial participation	N/A
Dissemination policy: trial results	#31a	Plans for investigators and sponsor to communicate trial results to participants, healthcare professionals, the public, and other relevant groups (eg, via publication, reporting in results databases, or other data sharing arrangements), including any publication restrictions	2
Dissemination policy: authorship	#31b	Authorship eligibility guidelines and any intended use of professional writers	N/A
Dissemination policy: reproducible research	#31c	Plans, if any, for granting public access to the full protocol, participant-level dataset, and statistical code	N/A
**Appendices**			
Informed consent materials	#32	Model consent form and other related documentation given to participants and authorised surrogates	N/A
Biological specimens	#33	Plans for collection, laboratory evaluation, and storage of biological specimens for genetic or molecular analysis in the current trial and for future use in ancillary studies, if applicable	7

The SPIRIT Explanation and Elaboration paper is distributed under the terms of the Creative Commons Attribution License CC-BY-NC. This checklist was completed on 04. March 2021 using https://www.goodreports.org/, a tool made by the EQUATOR Network in collaboration with Penelope.ai

For peer review only

BMJ Open

The SHAPER-PND trial: clinical effectiveness protocol of a community singing intervention for postnatal depression

Journal:	*BMJ Open*
Manuscript ID	bmjopen-2021-052133.R2
Article Type:	Protocol
Date Submitted by the Author:	05-Oct-2021
Complete List of Authors:	Estevao, Carolina; King's College London Institute of Psychiatry Psychology and Neuroscience, Department of Psychological Medicine<br>Bind, Rebecca; King’s College London, Department of Psychological Medicine<br>Fancourt, Daisy; University College London, Department of Behavioural Science and Health<br>Sawyer, Kristi; King’s College London, Department of Psychological Medicine<br>Dazzan, Paola; King's College London<br>Sevdalis, Nick; King's College London, Centre Implementation Science<br>Woods, Anthony; King’s College London, Department of Psychological Medicine<br>Crane, Nikki; King's College London<br>Rebecchini, Lavinia; King’s College London, Department of Psychological Medicine<br>Hazelgrove, Katie; King’s College London, Department of Psychological Medicine<br>Manoharan, Manonmani; South London and Maudsley NHS Foundation Trust<br>Burton, Alexandra; University College London, Department of Behavioural Science and Health<br>Dye, Hannah; Breathe Arts Health Research<br>Osborn, Tim; Breathe Arts Health Research<br>Davis, Rachel; King's College London, Centre for Implementation Science<br>Soukup, Tayana ; King's College London, Centre for Implementation Science<br>Arias de la Torre, Jorge; King's College London, Centre for Implementation Science; CIBER<br>Bakolis, Ioannis; King's College London, Centre for Implementation Science; King's College London, Department of Biostatistics and Health Informatics<br>Healey, Andy; King's College London, Health Service and Population Research Department<br>Perkins, Rosie; Royal College of Music, Centre for Performance Science; Imperial College London, Faculty of Medicine<br>Pariante, Carmine; King's College London, Psychological Medicine
**Primary Subject Heading**:	Mental health
Secondary Subject Heading:	Qualitative research, Research methods

Keywords:	MENTAL HEALTH, PSYCHIATRY, Depression & mood disorders < PSYCHIATRY, IMMUNOLOGY

SCHOLARONE™
Manuscripts

I, the Submitting Author has the right to grant and does grant on behalf of all authors of the Work (as defined in the below author licence), an exclusive licence and/or a non-exclusive licence for contributions from authors who are: i) UK Crown employees; ii) where BMJ has agreed a CC-BY licence shall apply, and/or iii) in accordance with the terms applicable for US Federal Government officers or employees acting as part of their official duties; on a worldwide, perpetual, irrevocable, royalty-free basis to BMJ Publishing Group Ltd ("BMJ") its licensees and where the relevant Journal is co-owned by BMJ to the co-owners of the Journal, to publish the Work in this journal and any other BMJ products and to exploit all rights, as set out in our licence.

The Submitting Author accepts and understands that any supply made under these terms is made by BMJ to the Submitting Author unless you are acting as an employee on behalf of your employer or a postgraduate student of an affiliated institution which is paying any applicable article publishing charge ("APC") for Open Access articles. Where the Submitting Author wishes to make the Work available on an Open Access basis (and intends to pay the relevant APC), the terms of reuse of such Open Access shall be governed by a Creative Commons licence – details of these licences and which Creative Commons licence will apply to this Work are set out in our licence referred to above.

Other than as permitted in any relevant BMJ Author's Self Archiving Policies, I confirm this Work has not been accepted for publication elsewhere, is not being considered for publication elsewhere and does not duplicate material already published. I confirm all authors consent to publication of this Work and authorise the granting of this licence.

For peer review only

BMJ OPEN

Title: The SHAPER-PND trial: clinical effectiveness protocol of a community singing intervention for postnatal depression

Authors: Carolina Estevao[1], Rebecca Bind[1], Daisy Fancourt[2], Kristi Sawyer[1], Paola Dazzan[1], Nick Sevdalis[3], Anthony Woods[1], Nikki Crane[4], Lavinia Rebecchini[1], Katie Hazelgrove[1], Manonmani Manoharan[5], Alexandra Burton[2], Hannah Dye[6], Tim Osborn[6], Rachel Davis[3], Tayana Soukup[3], Jorge Arias De La Torre[3,7], Ioannis Bakolis[3,8], Andy Healey[9], Rosie Perkins[10,11], Carmine M. Pariante[1]

[1]Department of Psychological Medicine, Institute of Psychiatry, Psychology and Neuroscience, King's College London, 5 Cutcombe Rd, Brixton, London SE5 9RT, United Kingdom

[2]Department of Behavioural Science and Health, University College London, Gower Street, London, WC1E 6BT, United Kingdom

[3]Centre for Implementation Science, Health Service and Population Research Department, Institute of Psychiatry, Psychology & Neuroscience, 16 De Crespigny Park, Camberwell, London SE5 8AB, United Kingdom

[4]Culture team, King's College London, Somerset House East Wing, Strand WC2R 2LS, United Kingdom

[5]South London and Maudsley NHS Foundation Trust, Denmark Hill, London SE5 8AZ, United Kingdom

[6]Breathe Arts Health Research, The Clarence Centre, 6 St George's Circus, London SE1 6FE, United Kingdom

[7]CIBER Epidemiology and Public Health (CIBERESP), Madrid, Spain

[8]Department of Biostatistics and Health Informatics, Institute of Psychiatry, Psychology & Neuroscience, 16 De Crespigny Park, Camberwell, London SE5 8AB, United Kingdom

[9]King's Health Economics, Health Service and Population Research Department, Institute of Psychiatry, Psychology & Neuroscience, 16 De Crespigny Park, Camberwell, London SE5 8AB, United Kingdom

[10]Centre for Performance Science, Royal College of Music, London, United Kingdom

[11]Faculty of Medicine, Imperial College London, London, United Kingdom

For peer review only

Corresponding author: Carolina Estevao, carolina.estevao@kcl.ac.uk

Keywords: arts intervention, randomised controlled trial, postnatal depression, mental health, singing

Word count: 3,973 words

Abstract

Introduction: Postnatal depression (PND) affects approximately 13% of new mothers. Community-based activities are sought after by many mothers, especially mothers that prefer not to access pharmacological or psychological interventions. Singing has shown positive effects in maternal mood and mother-child bonding. The Scaling-Up Health-Arts Programmes: Implementation and Effectiveness Research – Postnatal Depression (SHAPER-PND) study will analyse the clinical and implementation effectiveness of 10-week singing sessions for PND in new mothers. This protocol paper will focus on the clinical effectiveness of this trial.

Methods and analysis: A total of 400 mothers with PND (with a score of at least 10 on the Edinburgh Postnatal Depression Scale) and their babies will be recruited for this hybrid type II randomised controlled trial The intervention group will attend 10 weekly singing sessions held at community venues or online, facilitated by the arts organisation, Breathe Arts Health Research (Breathe). A control group will be encouraged to attend non-singing sessions in the community or online for 10 weeks. A package of assessments will be collected from participants for clinical, mechanistic and implementation outcomes, at different stages of the trial. Clinical assessments will include questionnaires and interviews for demographics, mental health, and social measures, together with biological samples for measurement of stress markers; the study visits are at baseline, week 6 (mid-trial) and week 10 (end of trial), with follow ups at weeks 20 and 36. Multiple Imputation will be used to deal with possible missing data and multivariable models will be fitted to assess differences between groups in the outcomes of the study.

Ethics and dissemination: Ethical approval has been granted by the London - West London & GTAC Research Ethics Committee, REC reference: 20/PR/0813. ClinicalTrials.gov Identifier: NCT04834622.

Article Summary

Strengths and limitations of this study

- Largest cohort of participants in a study examining singing in postnatal depression;

For peer review only

- Largest cohort studying biological measures in relation to an arts intervention for postnatal depression;
- Largest study to analyse the effect of an arts intervention on the mother-infant interaction;
- The state-of-the-art design will result in a unique package of clinical and implementation effectiveness on this intervention;
- There may be inconsistency throughout an intervention period if in-person sessions are switched online due to COVID-19 social distancing restrictions.

Introduction

Postnatal depression

Postnatal depression (PND) affects approximately 13% of new mothers, with symptoms including low mood, fatigue, anhedonia, insomnia and irritability [1,2]. Although pharmacological treatment has yielded positive results, these are hampered by low uptake and adherence, and while mother-infant psychotherapy has produced mixed results, poses similar challenges, including short-lived improvements [2–5].

Prior studies have found that PND carries implications for not only maternal health and wellbeing, but also for the mother-infant relationship [6]. A common finding is that, while interacting with their babies, depressed mothers spend more time in negative, withdrawn states [7,8] and exhibit reduced sensitivity [9,10], and in turn, their infants mirror this behaviour by disengaging and adopting a passive behavioural style [7]. Moreover, depressed mothers are more likely to report reduced feelings of attachment toward their infant [11] and are less likely to mentalize appropriately with their newborn [12]. Maternal sensitivity is highly predictive of infant secure attachment [13], which has long been linked to optimal developmental outcomes including mental health [14]; thus, intervention early-on to ameliorate the mother-infant interaction is warranted in order to protect offspring future development. However, while home-based interventions have been successful in improving PND, they show limited efficacy in helping the relationship itself [15]. Indeed, a recent combined psychoeducation and play intervention in a community setting was found to decrease dysfunctional parenting behaviours, but the intervention proved more effective in PND symptom reduction than in interaction improvement [16]; and a very small case study found that mother-infant painting sessions were successful in improving both the relationship and PND symptoms, though the sample size was only four dyads [17].

Despite these challenges in finding optimal treatment options, which can both alleviate maternal symptoms of PND and strengthen the mother-infant relationship, many mothers

For peer review only

continue to engage in community group activities, such as mother-infant play groups, as they are found to be effective at relaxing mothers, providing good sources of social interaction, decreasing the monotony of each day, and providing a sense of personal fulfilment for mothers [18]. Specifically, there is a growing body of evidence demonstrating the effects of community group singing on mental health [19,20] (see also the Melodies for Mums programme, described below). Singing to newborns is practised around the world, and research has demonstrated valuable benefits such as improving mother-infant interaction and reducing infant distress [21–23]. Listening to music during pregnancy is also associated with greater maternal wellbeing and reduced symptoms of PND in the first 3 months post-birth, and, additionally, daily singing to babies is associated with fewer symptoms of PND and increased maternal wellbeing, self-esteem and perceived mother-infant bond [24]. Consequently, there is a strong theoretical background for why singing could support mothers with PND. It is thought that postnatally depressed women may have difficulty with adherence to dyadic interventions given the nature and challenge of their symptoms [25]; however, no prior studies have assessed whether a community singing-based intervention may be able to both improve the quality of the developing relationship, as well as present as a more engaging intervention that mothers are likelier to complete.

Biological underpinning of singing sessions for postnatal depression

Three biological pathways are suggested to underlie the benefits of music engagement on mood: the glucocorticoid-modulated stress system, the inflammatory system, and the oxytocin system, which is also associated with maternal attachment and bonding. Of these, the hypothalamic-pituitary-adrenal (HPA) axis, of which the hormone cortisol is a main measure, is the most well-studied. Studies in various populations have given evidence for a reduction in salivary cortisol after a singing session [26-29], pointing to a consistent stress-reducing effect of singing. Interestingly, one of these studies also showed that singing sessions produced increases in the levels of cytokines such as IL-(interleukin-)17, IL-2, IL-4 and TNFα, which correlated with the observed reduction in cortisol [27]. The evidence surrounding the oxytocin system and music participation is more conflicted. Oxytocin is generally shown to increase as a result of singing [30,31], or even listening to music [32]. However, more recent studies have demonstrated a decrease in salivary oxytocin concentrations in certain circumstances, for example after choir singing, but not solo singing [28]. In addition, one study showed a decrease in oxytocin after singing sessions, which was associated with the increase in cytokines and reduction in cortisol, as described above [27]. This was proposed to be indicative of a general dampening of all stress-related signalling, in which oxytocin also plays a part, leading to an overall improved sense of wellbeing.

For peer review only

Studies investigating the interplay of these three biological systems in relation to musical engagement during the perinatal period are sparse. Indeed, only one study has investigated the biological effects of maternal singing groups [33], in which pregnant women were randomised to either music intervention, singing intervention, or control group. This study found a decrease in cortisol in both intervention groups, but that the effect was stronger in the singing group. Additionally, both musical intervention groups showed an increase in salivary oxytocin post-session. This is of particular interest given oxytocin's involvement in the mother-infant relationship [34], which may be strengthened in cases of mother-child biobehavioural synchrony, in which maternal and infant hormone levels covary [35].

The "Melodies for Mums" intervention

Melodies for Mums (M4M) is an intervention that was developed and tested as part of a collaboration between the Royal College of Music, Imperial College London and University College London (UK). M4M was tested in a three-arm randomised controlled trial (RCT) involving 134 mothers with PND (with an Edinburgh Postnatal Depression Scale (EPDS) score ≥ 10 at baseline, indicating depression), and compared the efficacy of community singing with a comparison group (10 weeks of creative play classes for dyads) and a wait-list control group (10 weeks of care-as-usual). This pilot study found that mothers with moderate-severe symptoms of PND, defined as an EPDS score of ≥13, who participated in the programme with their baby, had a significantly faster improvement in symptoms than mothers in usual care [36].

Following the pilot study, M4M was taken-on as a service by Breathe Arts Health Research (Breathe) across the boroughs of Lambeth and Southwark, in South London. However, there has since been no further research on the programme; thus, its effectiveness on a larger scale remains to be evaluated, as do the acceptability, appropriateness and feasibility of its delivery on a larger-scale, and its potential to be adopted and sustained as a cost-effective, beneficial service for mothers within national health services. In addition, there is no evidence on the effects of M4M on mother-infant interaction, and a detailed mechanistic investigation of the biological underpinning of any therapeutic effects is also missing. This paper will focus specifically on the protocol for a Wellcome-Trust-funded study aimed to deliver the clinical effectiveness and the biomarkers evaluation of M4M, the "Scaling-Up Health-Arts Programmes: Implementation and Effectiveness Research – Postnatal Depression" (SHAPER-PND) study. Information on the implementation effectiveness of M4M is discussed in a separate detailed protocol.

Methods and analysis

Aims

1) To determine the clinical effectiveness of the intervention in a larger sample size than previous studies (n= 400 women and their babies). This will allow us to ascertain whether the initial findings from the pilot study are replicable on a larger scale. To understand whether singing is an efficacious treatment for PND, including on mother-infant interaction, various psychological, social, and qualitative measures will be collected from women and their infants before, during, and after the intervention period.

2) To ascertain the biological mechanisms by which postnatal singing groups putatively improve maternal mood and mother-infant relationships. This will further the current understanding of the positive effects of the intervention and provide objectively measurable outcomes. Maternal and infant samples will be collected to measure cortisol and oxytocin, plus maternal inflammatory markers.

Objectives and outcomes

To address our aims, we have the following objectives and outcomes. As this protocol paper is focusing only on the clinical effectiveness of the trial, the objectives and outcomes of the implementation component of the trial have been reported in a separate manuscript.

Primary objective

1. Does community singing improve symptoms of PND?

Participants will complete the EPDS prior to the intervention (baseline) to ascertain the severity of their PND. The EPDS will be administered again at various timepoints throughout and following the intervention period (weeks 6, 10, 20 and 36).

Outcome: The primary outcome of this study is to understand whether singing reduces the severity of symptoms of PND on the EPDS between baseline and week 10 (end of intervention), via a reduction in total EPDS score.

Secondary Objectives

1. To assess whether singing improves (changes) further aspects of mental health, including depression using the Hamilton Depression Rating Scale (HDRS). Time Frame: Compare change between baseline and weeks 6, 10, 20 and 36.

2. To assess whether singing improves (changes) further aspects of mental health, including depression using the Beck Depression Inventory (BDI). Time Frame: Compare change between baseline and weeks 6, 10, 20 and 36.

For peer review only

3. To assess whether singing improves (changes) further aspects of mental health, including stress using the Perceived Stress Scale (PSS). Time Frame: Compare change between baseline and weeks 6, 10, 20 and 36.

4. To assess whether singing improves (changes) further aspects of mental health, including wellbeing using the Office for National Statistics Wellbeing Scale (ONS). Time Frame: Compare change between baseline and weeks 6, 10, 20 and 36.

5. To assess whether singing improves (changes) further aspects of mental health, including anxiety, using the State-Trait Anxiety Scale (STAI). Time Frame: Compare change between baseline and weeks 6, 10, 20 and 36.

6. To ascertain whether singing improves the observed mother-infant interaction using the Crittenden CARE-Index (CCI). Time Frame: Compare change between baseline and weeks 10 and 36.

7. To ascertain whether singing improves the perceived mother-infant relationship using the Maternal Postpartum Attachment Scale (MPAS). Time Frame: Compare change between baseline and weeks 6, 10, 20 and 36.

8. To ascertain whether singing improves the perceived mother-infant relationship using the Parent Reflective Functioning Questionnaire (PRFQ). Time Frame: Compare change between baseline and weeks 6, 10, 20 and 36.

9. To ascertain whether singing improves social support and reduces loneliness using the UCLA Loneliness Scale. Time Frame: Compare change between baseline and weeks 6, 10, 20 and 36.

10. To ascertain whether singing improves social support and reduces loneliness using the Multidimensional Scale of Perceived Social Support (MSPSS) Time Frame: Compare change between baseline and weeks 6, 10, 20 and 36.

11. To identify whether there are biological mechanisms underpinning the psychological outcomes assessed using changes in measurements in stress hormones, including hair cortisol, diurnal cortisol and salivary cytokines. Time Frame: Compare change between week 1 and weeks 6 and 10.

For peer review only

12. To identify whether there are biological mechanisms underpinning the psychological outcomes assessed using changes in measurements in salivary oxytocin. Time Frame: Compare change between week 1 and weeks 6 and 10.

13. To identify how the singing sessions affect the lived experience of mothers with PND using focus groups. Time Frame: Qualitative data collection at week 10 (end of intervention).

14. To explore the phenomenology of PND and how singing intersects with PND among women with particular risk factors for PND (traumatic birth, adverse childhood experiences, and social isolation/loneliness) using semi-structured interviews. Time Frame: Qualitative data collection at week 10 (end of intervention).

Data collection plan

The study is anticipated to run from September 2021 to December 2023.

In order to address the objectives within the clinical effectiveness arm of the study, data will be collected according to the data collection timeline in Table 1.

Table 1 Clinical effectiveness data collection timeline.

**Study objective**	**Type of measure**	**Measures**	**Timepoint collected**	**Type of data**
1	Mental health	Edinburgh Postnatal Depression Scale (EPDS)	Baseline, weeks 1, 6, 10, 20 & 36	Quantitative
2	Mental health	Hamilton Depression Rating Scale (HAM-D), Structured Clinical Interview for DSM-IV Disorders (SCID)	Weeks 1 & 10	Quantitative
		Beck Depression Inventory (BDI), State Trait Anxiety Inventory (STAI), Perceived Stress Scale (PSS); Office for	Weeks 1, 6, 10, 20 & 36	

For peer review only

		National Statistics Wellbeing Scale (ONS)		
3	Social	Maternal Postpartum Attachment Scale (MPAS), Parental Reflective Functioning Questionnaire (PRFQ)	Weeks 1, 6, 10, 20 & 36	Quantitative
		Crittenden Child-Adult Relationship (CCI CARE-Index)[1]	Baseline, weeks 10 & 36	
4	Social	UCLA Loneliness Scale, Short General Self-Efficacy Scale (GSE-6), Multidimensional Scale of Perceived Social Support (MSPSS)	Weeks 1, 6, 10, 20 & 36	Quantitative
5	Biological	Diurnal cortisol saliva samples[1]	Baseline & week 10	Quantitative
		Pre- and post-session saliva samples[1]	Weeks 1, 6, 10 (at sessions)	
		Hair samples	Week 10	
6	Qualitative	Focus groups with intervention groups to capture feedback from sessions and experiences from mothers in the groups, focusing on the lived experience of PND	Week 10	Qualitative
7	Qualitative	Semi-structured interviews with a sub sample of the intervention group of women self-reporting particular risk	Weeks 10-20, informed by information	Qualitative

For peer review only

		factors for PND (partially informed by demographic questionnaires)	collected at baseline	

[1] Collected from mothers and infants

In addition to the measures collected above, we will also collect comprehensive information on maternal and infant socio-demographics and medical health, maternal history of childhood maltreatment, and current maternal abuse, in order to explore the interaction between notable risk factors and the development of PND.

Trial Design

Scaling-Up Health-Arts Programmes: Implementation and Effectiveness Research (SHAPER) is a multi-disciplinary research programme funded by the Wellcome Trust that is hosted by King's College London (Sponsor) and University College London. The SHAPER-PND study described here is Hybrid Type II Effectiveness-Implementation randomised-controlled trial, where equal focus is placed on the effectiveness of the intervention and the effectiveness of its implementation [37].

Study Flowchart

An overview of the study timelines and procedures has been summarised in the flowchart below (

Figure 1):

For peer review only

Setting

This is a multi-centre trial that will be run in locations across London (primarily in the South London boroughs of Lewisham, Lambeth and Southwark), specifically in children's or community venues or online. These venues will be thoroughly safety- and risk-assessed in light of COVID-19 and adherence to any COVID-secure measures will be closely monitored by Breathe.

In order to enrol a sufficiently large sample of women, there will be 12 blocks of the 10-week singing programme in total: 10 intervention blocks will be for the experimental group, and 2 blocks will be for women in the wait-list control group that wish to take part in the singing sessions. The 2 wait-list blocks will be offered singing sessions after a woman has completed the first 10 weeks of the study and no data will be collected regarding these singing sessions, apart from the control follow-up data that this group is expected to provide.

In the event that one or several women need to self-isolate due to confirmed or suspected exposure to COVID-19, the sessions will be delivered to her/them online via Zoom. This will allow for the sessions to still be conducted in person with the remaining women.

In the event of the artist or the Breathe officer having to self-isolate, a replacement Breathe-trained artist will be ready to step-in and deliver the sessions. The same will apply to Breathe staff; there will be another staff member available to be present at the sessions.

Participants

Study participants will be new mothers exhibiting symptoms of PND, who meet criteria for a current major depressive episode, and have babies aged 0-9 months.

Inclusion Criteria: Women aged 18 or older; satisfactory understanding of English; women with infants aged 0-9 months; women with PND, who score ≥10 on the EPDS. Women will require access to an internet-connected device (mobile phone, tablet, computer or laptop) to allow completion of assessments and participation in the singing sessions in the event they go online.

Exclusion Criteria: The participant may not enter the study if her baby is outside of the age-range specified; if she is unable to give informed consent.

Number of participants and power analysis

The pilot RCT that informed this study showed that depressive symptoms (EPDS scores) improved in both singing and control women by the end of treatment (week 10), but the singing group had a numerically larger improvement (-5.2 (SD=2.8)) than the care-as-usual group (-

For peer review only

4.25 (SD=3.2), effect size=0.32)) [36]. To have power of 80% (at $p<0.05$, two-tailed) to detect the same effect size difference, randomising subjects 2:1, 232 subjects will be required in the intervention and 116 in the control group (total= 348). In total, 400 mothers and their babies will be recruited to compensate for a 12% drop-out rate.

Participants will be recruited in blocks of 40-50 women and their babies, screened and assessed, prior to a 2:1 allocation randomisation (dyads will be allocated the same group). This process will be repeated 12 times, aiming to reach 400 dyads.

Recruitment process

The recruitment period will last two years (2021-2022). All potential participants will initially be directed to a pre-screening online form that includes an EPDS. If the EPDS score is < 10, a participant will be notified that she is not currently eligible to participate and will be signposted to other support services within the community (e.g., talking therapies, mother-baby groups, baby activity groups, etc). If a woman's score is ≥ 10, she will be notified that she is potentially eligible and will proceed straight to the second phase of screening through a home (or Zoom) visit. The participant information sheet (PIS) for the trial will be sent once the baseline assessment is booked.

When a new round of 10 classes becomes available, researchers will arrange assessments with potential participants to undertake the full screening interview against the inclusion/exclusion criteria. If the participant is found non-eligible, she will be signposted to other support services within the community.

The singing group

M4M is a 10-week intervention for mothers with PND. The programme will be delivered to groups of 8–12 mothers in weekly sessions lasting one hour. Mothers will start a block of 10-week classes together and continue with the same group and leader for the duration of the course. Classes are free to attend and will take place in Children's Centres or other community venues (or online via Zoom). Mothers will attend with their babies and will be invited to sit in a socially distanced circle on the floor surrounded by soft play cushions and mats. Classes will start with welcome songs, introducing the babies and mothers to one another, and then involve a range of singing and music activities. Mothers will be required to respect social distancing guidelines and keep their babies within their reach to avoid physical contact with other mothers or their babies.

Some songs will be accompanied by maracas, drums, hand chimes and other simple instruments that the mothers and babies can play together. Instruments such as guitar and ukulele will also be used for a small number of songs. Instruments will not be shared between

For peer review only

the participants and will be disinfected before and after the singing sessions with a COVID-19 validated disinfectant.

Mothers will also work to write some of their own songs over the weeks, developing lyrics together about their babies or experiences of motherhood and creating simple melodies. Recordings of the group singing the songs together will be made and uploaded to private online platforms or onto CDs for the mothers to listen to at home. Classes will be led by professional workshop leaders trained by Breathe, with support of assistants.

The control group

The control group will be an 'active' control. During the first 10 weeks (during the study period), mothers in the control group will receive details of other non-music classes available to them in the community (either in-person or online, depending on the programs available at the time and government guidelines) and will receive the same schedule of texts and phone calls to encourage them to join these activities. They will still be seen by the researchers to collect clinical measures and biological samples (including the pre-post saliva samples) and to monitor engagement in other activities. Following the first 10 weeks, the mothers in the control group will be offered a place on the singing programme, but these data will not be part of the study, and they will not join groups with other women who are in the study.

Patient and public involvement

Participants from previous sessions delivered by Breathe in the community have provided valuable feedback and insights into the sessions' delivery, content and schedule. These were gathered through focus groups and workshops where mothers and others involved in the singing sessions were able to share their experience of the sessions. This body of work allowed the refinement of the sessions for the intervention to be more suitable for the population.

A previous RCT was designed and delivered by two of this study's co-authors (DF, RP), along with Breathe [36]. The RCT analysed the clinical efficacy of the intervention on a smaller scale and explored the experiences of participants receiving and delivering the intervention, which ultimately informed the design of this SHAPER-PND study.

Data Analyses

Quantitative analyses

The main statistical analyses will be carried out by the trial statistician who will be blind to the group randomisation until the main analyses are complete. The analyses outlined in this

For peer review only

strategy will be primarily based on intention to treat. A per-protocol analysis will also be conducted in which the primary outcome will also be compared between groups, removing data of participants who:

1. Do not complete their intervention as defined above. 2. Miss over 50% of the singing or control sessions. 3. Are later found to have an exclusion criterion that was missed.

The first stage of analyses will be a descriptive model of the data to assess their completeness. Initially, a complete case analysis was performed under a missing at random assumption (MAR) where explanatory variables could predict the missing values in the respective outcome variable. These models drop any participant who does not provide outcome data at the follow up time point.

Mother-level baseline variables will be described both overall and by randomised groups. Patterns of missing data will be described, if any. predictors of missingness will be also assessed. The primary outcome will be analysed using linear mixed models to model the change in total score of EPDS at 6-, 10-, 20- and 36-weeks post randomisation. A three-level hierarchical model will be employed with all-time points included as repeated measures in the model (6-, 10-, 20- and 36-weeks post randomisation) adjusting for baseline EPDS to improve power and take into account clustering of the observation at mother's level. Associations between post randomisation variables and missingness will be dealt with by multiple imputation by chained equations, again under the MAR assumption. Departures from this assumption will be assessed with a sensitivity analysis. All baseline characteristics as well as the outcomes will be included in the MI models. Secondary outcomes (e.g., PSS, HAM-D) will be assessed with a similar methodology for the primary outcomes, using generalised linear mixed models depending on the type of outcome (normal, binary and count). The questionnaires to be used have validated methods of scoring, and the scores will be analysed as described. All analyses will be conducted in STATA V.15.1.

Qualitative analyses

Data from the focus groups will be analysed using Thematic Analysis [38], to understand the lived experience of PND and how this relates to experiences in the singing group. Data from the individual/small-group interviews with sub-groups of women self-reporting particular risk factors for PND (traumatic birth, adverse childhood experiences, and social isolation/loneliness) will be analysed using Interpretative Phenomenological Analysis (IPA) [39] to focus in-depth on how singing intersects with the specific context of PND among women in these sub-groups.

Ethics and dissemination

For peer review only

Ethical approval has been granted by the London - West London & GTAC Research Ethics Committee, REC reference: 20/PR/0813.

Samples will be handled according to HTA regulations. All data will be handled in line with GDPR and other relevant regulations.

Informed consent will be collected from all research participants and stakeholders involved in the study.

Findings will be published in peer-reviewed journals and disseminated at national and international meetings. Participants will be made aware of the results of the study through a newsletter.

ClinicalTrials.gov Identifier: NCT04834622

Contributorship statement

All authors listed have contributed to the conception and design of the protocol and this manuscript. All authors have been involved in the drafting of the manuscript and have individually approved the version of the work published.
Specifically, the contribution of each author falls within the following CRediT categories:
CE, RB, KS, PD, NS, LR, KH, AB, RD, TS, JAT, IB and AH : conceptualization, methodology and project administration.

DF, RP, CP: conceptualisation, methodology, supervision and funding acquisition.

NC, AW: conceptualisation, project administration and funding acquisition.

MM, HD and TO: conceptualisation, project administration.

Competing interests

NS is the director of the London Safety and Training Solutions Ltd, which offers training in patient safety, implementation solutions and human factors to healthcare organisations.

DF is a non-executive board director for Breathe Arts Health Research but she receives no financial compensation for her involvement.

TS received funding for training cancer multidisciplinary teams in the assessment and quality improvement methods in the United Kingdom. TS serves as a consultant to F. Hoffmann-La

For peer review only

Roche Diagnostics providing advisory research services in relation to innovations for cancer multidisciplinary teams and their care planning meetings in the United States of America.

CMP has received research and consultation funding from Boehringer Ingelheim and Johnson & Johnson for research on depression and inflammation, and by a Wellcome Trust strategy award to the Neuroimmunology of Mood Disorders and Alzheimer's Disease (NIMA) Consortium (104025), which is also funded by Janssen, GlaxoSmithKline, Lundbeck and Pfizer; the work presented in this paper is unrelated to this funding.

The other authors have no conflicts of interest to declare.

Funding Statement

This trial is part of the SHAPER programme, a Scaling-up Health-Arts Programme to scale up arts interventions. This programme is funded by the Wellcome Trust (award reference 219425/Z/19/Z). This work is additionally supported by the National Institute for Health Research (NIHR) Biomedical Research Centre at South London and Maudsley NHS Foundation Trust and King's College London and by a NIHR Senior Investigator to CMP.

NS, IB and AH' research is supported by the National Institute for Health Research (NIHR) Applied Research Collaboration (ARC) South London at King's College Hospital NHS Foundation Trust. NS and AH are members of King's Improvement Science, which offers co-funding to the NIHR ARC South London and comprises a specialist team of improvement scientists and senior researchers based at King's College London. Its work is funded by King's Health Partners (Guy's and St Thomas' NHS Foundation Trust, King's College Hospital NHS Foundation Trust, King's College London and South London and Maudsley NHS Foundation Trust), Guy's and St Thomas' Charity and the Maudsley Charity. The views expressed in this publication are those of the authors and not necessarily those of the Wellcome Trust, the NIHR, or the Department of Health and Social Care.

RD is supported by the National Institute for Health Research (NIHR) Applied Research Collaboration: South London, at King's College Hospital NHS Foundation Trust. The views expressed in this publication are those of the author(s) and not necessarily those of the NHS, NIHR or the Department of Health and Social Care.

Data sharing statement: Not applicable, this manuscript does not contain any data.

References

1 Boath E, Henshaw C. The treatment of postnatal depression: A comprehensive literature review. *J Reprod Infant Psychol* 2001;**19**:215–48. doi:10.1080/02646830120073224

For peer review only

2 Morrell C, Warner R, Slade P, *et al.* Psychological interventions for postnatal depression: cluster randomised trial and economic evaluation. The PoNDER trial. *Health Technol Assess* 2009;**13**. doi:10.3310/hta13300

3 Bledsoe SE, Grote NK. Treating Depression During Pregnancy and the Postpartum: A Preliminary Meta-Analysis. *Res Soc Work Pract* 2006;**16**:109–20. doi:10.1177/1049731505282202

4 Cooper PJ, Murray L, Wilson A, *et al.* Controlled trial of the short- and long-term effect of psychological treatment of post-partum depression 1. Impact on maternal mood. *Br J Psychiatry* 2003;**182**:412–9. doi:10.1192/bjp.182.5.412

5 Milgrom J, Negri LM, Gemmill AW, *et al.* A randomized controlled trial of psychological interventions for postnatal depression. *Br J Clin Psychol Br Psychol Soc* 2005;**44**:529–42. doi:10.1348/014466505X34200

6 Murray L, Fearon P, Cooper P. Postnatal depression, mother-infant interactions, and child development: Prospects for screening and treatment. In: *Identifying perinatal depression and anxiety: Evidence-based practice in screening, psychosocial assessment, and management.* Wiley-Blackwell 2015. 139–64.

7 Field T, Healy BT, Goldstein S, *et al.* Behavior-state matching and synchrony in mother-infant interactions of nondepressed versus depressed dyads. *Dev Psychol* 1990;**26**:7–14. doi:10.1037/0012-1649.26.1.7

8 Field T. Maternal Depression Effects on Infants and Early Interventions. *Prev Med* 1998;**27**:200–3. doi:10.1006/pmed.1998.0293

9 Field T. Postpartum depression effects on early interactions, parenting, and safety practices: A review. *Infant Behav Dev* 2010;**33**:1–6. doi:10.1016/j.infbeh.2009.10.005

10 Murray L, Fiori-Cowley A, Hooper R, *et al.* The impact of postnatal depression and associated adversity on early mother-infant interactions and later infant outcome. *Child Dev* 1996;**67**:2512–26.

11 Moehler E, Brunner R, Wiebel A, *et al.* Maternal depressive symptoms in the postnatal period are associated with long-term impairment of mother–child bonding. *Arch Womens Ment Health* 2006;**9**:273–8. doi:10.1007/s00737-006-0149-5

12 Pawlby S, Fernyhough C, Meins E, *et al.* Mind-mindedness and maternal responsiveness in infant–mother interactions in mothers with severe mental illness. *Psychol Med* 2010;**40**:1861–9. doi:10.1017/S0033291709992340

13 Campbell SB, Brownell CA, Hungerford A, *et al.* The course of maternal depressive symptoms and maternal sensitivity as predictors of attachment security at 36 months. *Dev Psychopathol* 2004;**16**. doi:10.1017/S0954579404044499

14 DeKlyen M, Greenberg M. Attachment and psychopathology in childhood. In: *Handbook of attachment: Theory, research, and clinical applications*. The Guilford Press 2008. 637–65.

15 Gelfand DM, Teti DM, Seiner SA, *et al.* Helping mothers fight depression: Evaluation of a home-based intervention program for depressed mothers and their infants. *J Clin Child Psychol* 1996;**25**:406–22. doi:10.1207/s15374424jccp2504_6

For peer review only

16 Ericksen J, Loughlin E, Holt C, *et al.* A therapeutic playgroup for depressed mothers and their infants: feasibility study and pilot randomized trial of community hugs. *Infant Ment Health J* 2018;**39**:396–409. doi:10.1002/imhj.21723

17 Arroyo C, Fowler N. Before and after: A mother and infant painting group. *Int J Art Ther* 2013;**18**:98–112. doi:10.1080/17454832.2013.844183

18 Feeley N, Bell L, Hayton B, *et al.* Care for Postpartum Depression: What Do Women and Their Partners Prefer? *Perspect Psychiatr Care* 2016;**52**:120–30. doi:10.1111/ppc.12107

19 Clift S, Morrison I. Group singing fosters mental health and wellbeing: findings from the East Kent “singing for health” network project. *Ment Health Soc Incl* 2011;**15**:88–97. doi:10.1108/20428301111140930

20 Coulton S, Clift S, Skingley A, *et al.* Effectiveness and cost-effectiveness of community singing on mental health-related quality of life of older people: randomised controlled trial. *Br J Psychiatry J Ment Sci* 2015;**207**:250–5. doi:10.1192/bjp.bp.113.129908

21 Mualem O, Klein PS. The communicative characteristics of musical interactions compared with play interactions between mothers and their one-year-old infants. *Early Child Dev Care* 2013;**183**:899–915. doi:10.1080/03004430.2012.688824

22 Nakata T, Trehub SE. Infants’ responsiveness to maternal speech and singing. *Infant Behav Dev* 2004;**27**:455–64. doi:10.1016/j.infbeh.2004.03.002

23 Vlismas W, Malloch S, Burnham D. The effects of music and movement on mother–infant interactions. *Early Child Dev Care* 2013;**183**:1669–88. doi:10.1080/03004430.2012.746968

24 Fancourt D, Perkins R. Associations between singing to babies and symptoms of postnatal depression, wellbeing, self-esteem and mother-infant bond. *Public Health* 2017;**145**:149–52. doi:10.1016/j.puhe.2017.01.016

25 Nylen KJ, Moran TE, Franklin CL, *et al.* Maternal depression: A review of relevant treatment approaches for mothers and infants. *Infant Ment Health J* 2006;**27**:327–43. doi:10.1002/imhj.20095

26 Fancourt D, Aufegger L, Williamon A. Low-stress and high-stress singing have contrasting effects on glucocorticoid response. *Front Psychol* 2015;**6**. doi:10.3389/fpsyg.2015.01242

27 Fancourt D, Williamon A, Carvalho LA, *et al.* Singing modulates mood, stress, cortisol, cytokine and neuropeptide activity in cancer patients and carers. *ecancermedicalscience* 2016;**10**. doi:10.3332/ecancer.2016.631

28 Schladt TM, Nordmann GC, Emilius R, *et al.* Choir versus Solo Singing: Effects on Mood, and Salivary Oxytocin and Cortisol Concentrations. *Front Hum Neurosci* 2017;**11**:430. doi:10.3389/fnhum.2017.00430

29 Grebosz-Haring K, Thun-Hohenstein L. Effects of group singing versus group music listening on hospitalized children and adolescents with mental disorders: A pilot study. *Heliyon* 2018;**4**:e01014. doi:10.1016/j.heliyon.2018.e01014

30 Grape C, Sandgren M, Hansson L-O, *et al.* Does singing promote well-being?: An empirical study of professional and amateur singers during a singing lesson. *Integr Physiol Behav Sci* 2002;**38**:65–74. doi:10.1007/BF02734261

For peer review only

31 Kreutz G. Does singing promote social bonding? *Music Med* 2014;**6**:51–60.

32 Nilsson U. Soothing music can increase oxytocin levels during bed rest after open-heart surgery: a randomised control trial. *J Clin Nurs* 2009;**18**:2153–61. doi:10.1111/j.1365-2702.2008.02718.x

33 Wulff V, Hepp P, Wolf OT, *et al.* The effects of a music and singing intervention during pregnancy on maternal well-being and mother–infant bonding: a randomised, controlled study. *Arch Gynecol Obstet* Published Online First: 10 August 2020. doi:10.1007/s00404-020-05727-8

34 Galbally M, Lewis AJ, van IJzendoorn M, *et al.* The Role of Oxytocin in Mother-Infant Relations: A Systematic Review of Human Studies: *Harv Rev Psychiatry* 2011;**19**:1–14. doi:10.3109/10673229.2011.549771

35 Feldman R. Oxytocin and social affiliation in humans. *Horm Behav* 2012;**61**:380–91. doi:10.1016/j.yhbeh.2012.01.008

36 Fancourt D, Perkins R. Effect of singing interventions on symptoms of postnatal depression: three-arm randomised controlled trial. *Br J Psychiatry* 2018;**212**:119–21. doi:10.1192/bjp.2017.29

37 Curran GM, Bauer M, Mittman B, *et al.* Effectiveness-implementation hybrid designs: combining elements of clinical effectiveness and implementation research to enhance public health impact. *Med Care* 2012;**50**:217–26. doi:10.1097/MLR.0b013e3182408812

38 Smith JA. *Qualitative Psychology: A Practical Guide to Research Methods*. SAGE 2015.

39 Smith JA, Shinebourne P. Interpretative phenomenological analysis. In: *APA handbook of research methods in psychology, Vol 2: Research designs: Quantitative, qualitative, neuropsychological, and biological*. Washington, DC, US: : American Psychological Association 2012. 73–82. doi:10.1037/13620-005

For peer review only

Figure legend:

Figure 1 SHAPER-PND study flowchart.

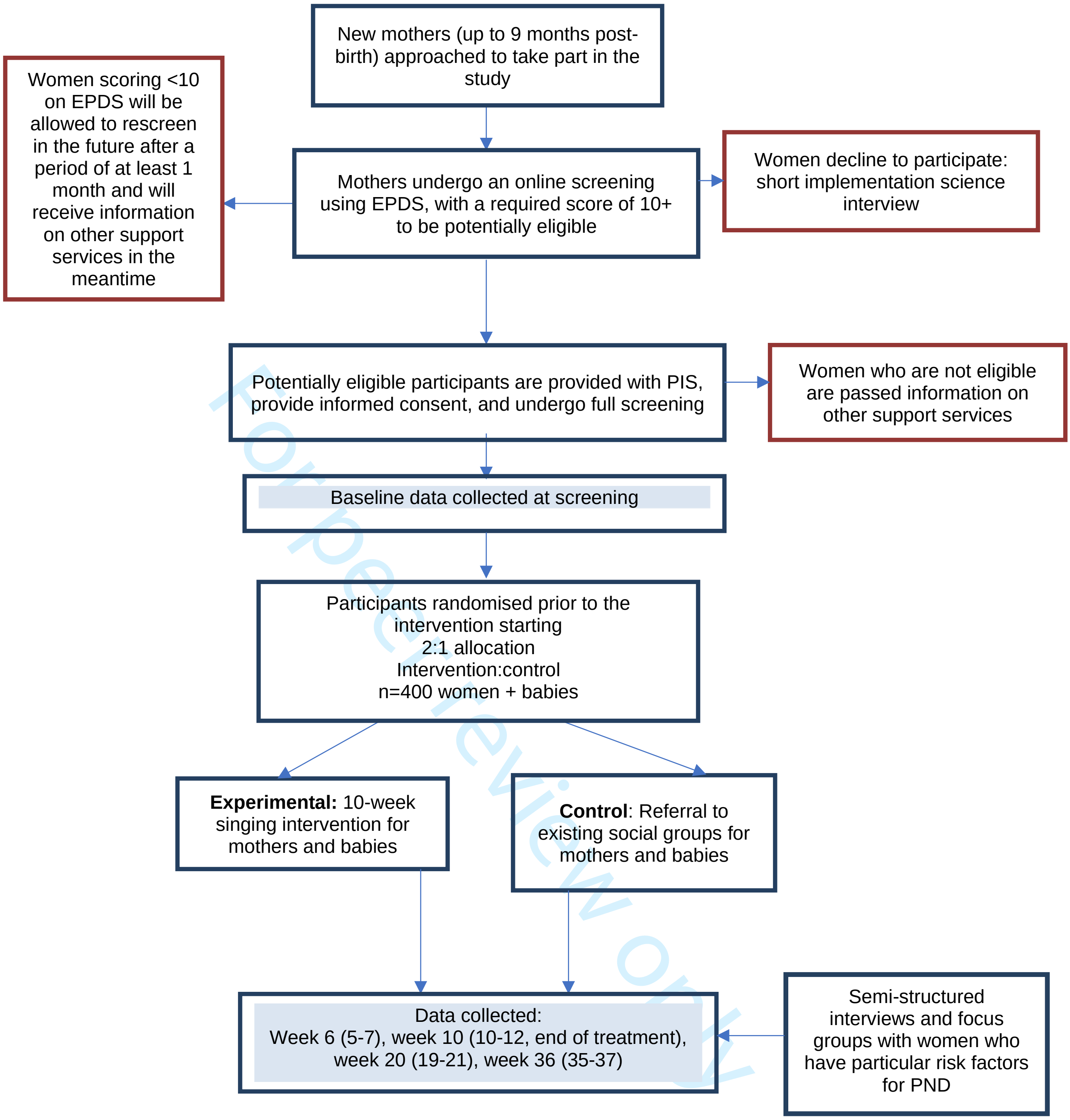
New mothers (up to 9 months post-birth) approached to take part in the study
Women scoring <10 on EPDS will be allowed to rescreen in the future after a period of at least 1 month and will receive information on other support services in the meantime
Mothers undergo an online screening using EPDS, with a required score of 10+ to be potentially eligible
Women decline to participate: short implementation science interview
Potentially eligible participants are provided with PIS, provide informed consent, and undergo full screening
Women who are not eligible are passed information on other support services
Baseline data collected at screening
Participants randomised prior to the intervention starting
2:1 allocation
Intervention:control
n=400 women + babies
Experimental: 10-week singing intervention for mothers and babies
Control: Referral to existing social groups for mothers and babies
Data collected:
Week 6 (5-7), week 10 (10-12, end of treatment), week 20 (19-21), week 36 (35-37)
Semi-structured interviews and focus groups with women who have particular risk factors for PND

Reporting checklist for protocol of a clinical trial.

Based on the SPIRIT guidelines.

Instructions to authors

Complete this checklist by entering the page numbers from your manuscript where readers will find each of the items listed below.

Your article may not currently address all the items on the checklist. Please modify your text to include the missing information. If you are certain that an item does not apply, please write "n/a" and provide a short explanation.

Upload your completed checklist as an extra file when you submit to a journal.

In your methods section, say that you used the SPIRITreporting guidelines, and cite them as:

Chan A-W, Tetzlaff JM, Gøtzsche PC, Altman DG, Mann H, Berlin J, Dickersin K, Hróbjartsson A, Schulz KF, Parulekar WR, Krleža-Jerić K, Laupacis A, Moher D. SPIRIT 2013 Explanation and Elaboration: Guidance for protocols of clinical trials. BMJ. 2013;346:e7586

		Reporting Item	Page Number
**Administrative information**			
Title	#1	Descriptive title identifying the study design, population, interventions, and, if applicable, trial acronym	1
Trial registration	#2a	Trial identifier and registry name. If not yet registered, name of intended registry	1
Trial registration: data set	#2b	All items from the World Health Organization Trial Registration Data Set	1-14
Protocol version	#3	Date and version identifier	1
Funding	#4	Sources and types of financial, material, and other support	19-20
Roles and responsibilities: contributorship	#5a	Names, affiliations, and roles of protocol contributors	1

For peer review only

Roles and responsibilities: sponsor contact information	#5b	Name and contact information for the trial sponsor	9
Roles and responsibilities: sponsor and funder	#5c	Role of study sponsor and funders, if any, in study design; collection, management, analysis, and interpretation of data; writing of the report; and the decision to submit the report for publication, including whether they will have ultimate authority over any of these activities	9
Roles and responsibilities: committees	#5d	Composition, roles, and responsibilities of the coordinating centre, steering committee, endpoint adjudication committee, data management team, and other individuals or groups overseeing the trial, if applicable (see Item 21a for data monitoring committee)	N/A
**Introduction**			
Background and rationale	#6a	Description of research question and justification for undertaking the trial, including summary of relevant studies (published and unpublished) examining benefits and harms for each intervention	3-5
Background and rationale: choice of comparators	#6b	Explanation for choice of comparators	9
Objectives	#7	Specific objectives or hypotheses	6-7
Trial design	#8	Description of trial design including type of trial (eg, parallel group, crossover, factorial, single group), allocation ratio, and framework (eg, superiority, equivalence, non-inferiority, exploratory)	9
**Methods: Participants, interventions, and outcomes**			
Study setting	#9	Description of study settings (eg, community clinic, academic hospital) and list of countries where data will be collected. Reference to where list of study sites can be obtained	11
Eligibility criteria	#10	Inclusion and exclusion criteria for participants. If applicable, eligibility criteria for study centres and individuals who will	12

For peer review only

		perform the interventions (eg, surgeons, psychotherapists)	
Interventions: description	#11a	Interventions for each group with sufficient detail to allow replication, including how and when they will be administered	5
Interventions: modifications	#11b	Criteria for discontinuing or modifying allocated interventions for a given trial participant (eg, drug dose change in response to harms, participant request, or improving / worsening disease)	N/A
Interventions: adherance	#11c	Strategies to improve adherence to intervention protocols, and any procedures for monitoring adherence (eg, drug tablet return; laboratory tests)	N/A
Interventions: concomitant care	#11d	Relevant concomitant care and interventions that are permitted or prohibited during the trial	N/A
Outcomes	#12	Primary, secondary, and other outcomes, including the specific measurement variable (eg, systolic blood pressure), analysis metric (eg, change from baseline, final value, time to event), method of aggregation (eg, median, proportion), and time point for each outcome. Explanation of the clinical relevance of chosen efficacy and harm outcomes is strongly recommended	14
Participant timeline	#13	Time schedule of enrolment, interventions (including any run-ins and washouts), assessments, and visits for participants. A schematic diagram is highly recommended (see Figure)	10
Sample size	#14	Estimated number of participants needed to achieve study objectives and how it was determined, including clinical and statistical assumptions supporting any sample size calculations	6
Recruitment	#15	Strategies for achieving adequate participant enrolment to reach target sample size	12
**Methods: Assignment of interventions (for controlled trials)**			
Allocation: sequence generation	#16a	Method of generating the allocation sequence (eg, computer-generated random numbers), and list of any factors for stratification. To reduce predictability of a random sequence, details of any planned restriction (eg, blocking) should be provided in a separate document that is unavailable to those who enrol participants or assign interventions	N/A

For peer review only

Allocation concealment mechanism	#16b	Mechanism of implementing the allocation sequence (eg, central telephone; sequentially numbered, opaque, sealed envelopes), describing any steps to conceal the sequence until interventions are assigned	N/A
Allocation: implementation	#16c	Who will generate the allocation sequence, who will enrol participants, and who will assign participants to interventions	N/A
Blinding (masking)	#17a	Who will be blinded after assignment to interventions (eg, trial participants, care providers, outcome assessors, data analysts), and how	N/A
Blinding (masking): emergency unblinding	#17b	If blinded, circumstances under which unblinding is permissible, and procedure for revealing a participant's allocated intervention during the trial	N/A
**Methods: Data collection, management, and analysis**			
Data collection plan	#18a	Plans for assessment and collection of outcome, baseline, and other trial data, including any related processes to promote data quality (eg, duplicate measurements, training of assessors) and a description of study instruments (eg, questionnaires, laboratory tests) along with their reliability and validity, if known. Reference to where data collection forms can be found, if not in the protocol	7
Data collection plan: retention	#18b	Plans to promote participant retention and complete follow-up, including list of any outcome data to be collected for participants who discontinue or deviate from intervention protocols	N/A
Data management	#19	Plans for data entry, coding, security, and storage, including any related processes to promote data quality (eg, double data entry; range checks for data values). Reference to where details of data management procedures can be found, if not in the protocol	N/A
Statistics: outcomes	#20a	Statistical methods for analysing primary and secondary outcomes. Reference to where other details of the statistical analysis plan can be found, if not in the protocol	13-14
Statistics: additional analyses	#20b	Methods for any additional analyses (eg, subgroup and adjusted analyses)	N/A

For peer review only

Statistics: analysis population and missing data	#20c	Definition of analysis population relating to protocol non-adherence (eg, as randomised analysis), and any statistical methods to handle missing data (eg, multiple imputation)	13-14
**Methods: Monitoring**			
Data monitoring: formal committee	#21a	Composition of data monitoring committee (DMC); summary of its role and reporting structure; statement of whether it is independent from the sponsor and competing interests; and reference to where further details about its charter can be found, if not in the protocol. Alternatively, an explanation of why a DMC is not needed	N/A
Data monitoring: interim analysis	#21b	Description of any interim analyses and stopping guidelines, including who will have access to these interim results and make the final decision to terminate the trial	N/A
Harms	#22	Plans for collecting, assessing, reporting, and managing solicited and spontaneously reported adverse events and other unintended effects of trial interventions or trial conduct	N/A
Auditing	#23	Frequency and procedures for auditing trial conduct, if any, and whether the process will be independent from investigators and the sponsor	N/A
**Ethics and dissemination**			
Research ethics approval	#24	Plans for seeking research ethics committee / institutional review board (REC / IRB) approval	1
Protocol amendments	#25	Plans for communicating important protocol modifications (eg, changes to eligibility criteria, outcomes, analyses) to relevant parties (eg, investigators, REC / IRBs, trial participants, trial registries, journals, regulators)	N/A
Consent or assent	#26a	Who will obtain informed consent or assent from potential trial participants or authorised surrogates, and how (see Item 32)	2
Consent or assent: ancillary studies	#26b	Additional consent provisions for collection and use of participant data and biological specimens in ancillary studies, if applicable	2
Confidentiality	#27	How personal information about potential and enrolled participants will be collected, shared, and maintained in order to protect confidentiality before, during, and after the trial	N/A

For peer review only

Declaration of interests	#28	Financial and other competing interests for principal investigators for the overall trial and each study site	19
Data access	#29	Statement of who will have access to the final trial dataset, and disclosure of contractual agreements that limit such access for investigators	N/A
Ancillary and post trial care	#30	Provisions, if any, for ancillary and post-trial care, and for compensation to those who suffer harm from trial participation	N/A
Dissemination policy: trial results	#31a	Plans for investigators and sponsor to communicate trial results to participants, healthcare professionals, the public, and other relevant groups (eg, via publication, reporting in results databases, or other data sharing arrangements), including any publication restrictions	2
Dissemination policy: authorship	#31b	Authorship eligibility guidelines and any intended use of professional writers	N/A
Dissemination policy: reproducible research	#31c	Plans, if any, for granting public access to the full protocol, participant-level dataset, and statistical code	N/A
**Appendices**			
Informed consent materials	#32	Model consent form and other related documentation given to participants and authorised surrogates	N/A
Biological specimens	#33	Plans for collection, laboratory evaluation, and storage of biological specimens for genetic or molecular analysis in the current trial and for future use in ancillary studies, if applicable	7

The SPIRIT Explanation and Elaboration paper is distributed under the terms of the Creative Commons Attribution License CC-BY-NC. This checklist was completed on 04. March 2021 using https://www.goodreports.org/, a tool made by the EQUATOR Network in collaboration with Penelope.ai

For peer review only
